# Supplementary material for: A simplified co-culture reveals altered cardiotoxic responses to doxorubicin in hPSC-derived cardiomyocytes in the presence of endothelial cells
Source: Stem Cell Reports. 2026 Feb 12;21(3):102816. doi: 10.1016/j.stemcr.2026.102816 (PMC12985372; doi:10.1016/j.stemcr.2026.102816)
Supplement: Document S1. Figures S1–S5, Tables S1 and S2, and supplemental methods [file mmc1.pdf]

Stem Cell Reports, Volume *21*

Supplemental Information

A simplified co-culture reveals altered cardiotoxic responses to doxorubicin in hPSC-derived cardiomyocytes in the presence of endothelial cells

Marcella Brescia, James Gallant, Andrea Chatrian, Paul Keselman, Elsa Sörman Paulsson, Mervyn P.H. Mol, Rickard Sjögren, Karine Raymond, Valeria Orlova, Kalpana Barnes, Richard Wales, Jonas Austerjost, Michael W. Olszowy, Christine L. Mummery, Berend J. van Meer, and Richard P. Davis

a)

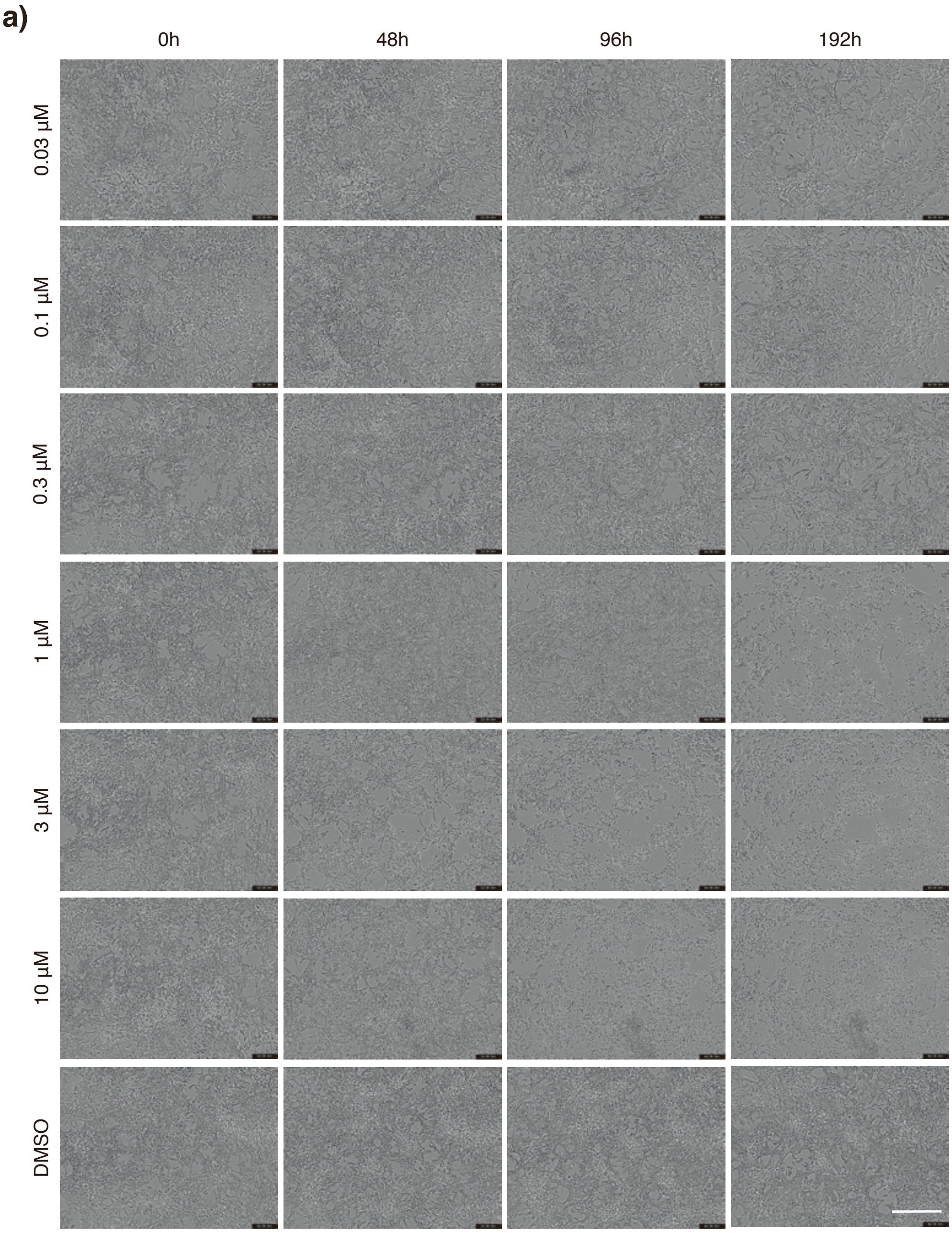
0h
48h
96h
192h
0.03 µM
0.1 µM
0.3 µM
1 µM
3 µM
10 µM
DMSO

b)

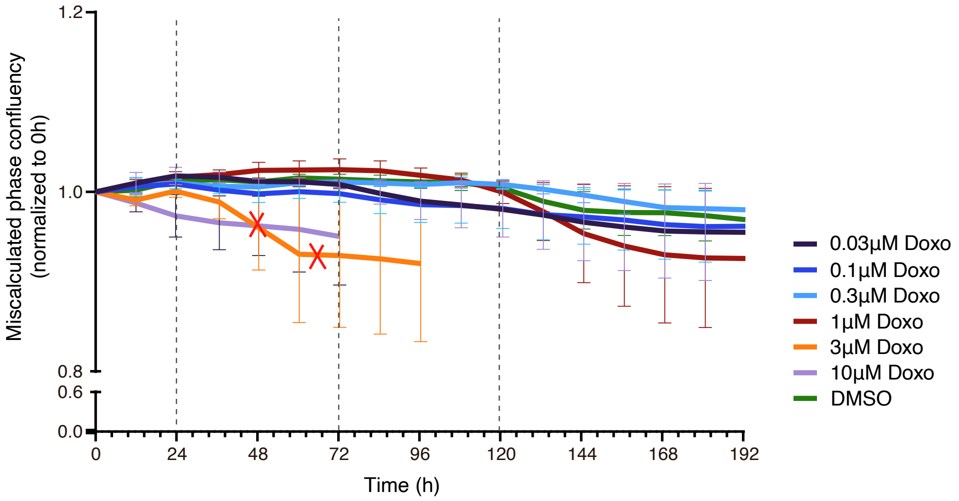
Miscalculated phase confluency (normalized to 0h)
1.2
1.0
0.8
0.6
0.0
0
24
48
72
96
120
144
168
192
Time (h)
0.03µM Doxo
0.1µM Doxo
0.3µM Doxo
1µM Doxo
3µM Doxo
10µM Doxo
DMSO

Figure S1. Effect of cumulative Doxo treatment on hiPSC-CMs, related to Figure 1.

a) Representative phase contrast images of hiPSC-CM monocultures treated cumulatively with various Doxo concentrations or DMSO (vehicle control). Images were acquired at baseline (0h), 24 h after the first and second treatments (48h and 96h, respectively), and at the final time point (192h). All treatments followed the cumulative dosing protocol outlined in Fig. 1, except 10 μM Doxo, which was administered as a single exposure. Scale bar, 400μm.

b) Incucyte-based quantification of phase confluency (percentage of the image area covered by objects), normalized to baseline (0 h). Red crosses indicate time points at which all cells were visually assessed as non-viable. Dotted lines indicate Doxo treatment time points. Data represent mean ± SEM (*n* = 3 biological replicates, each with 3 technical replicates).

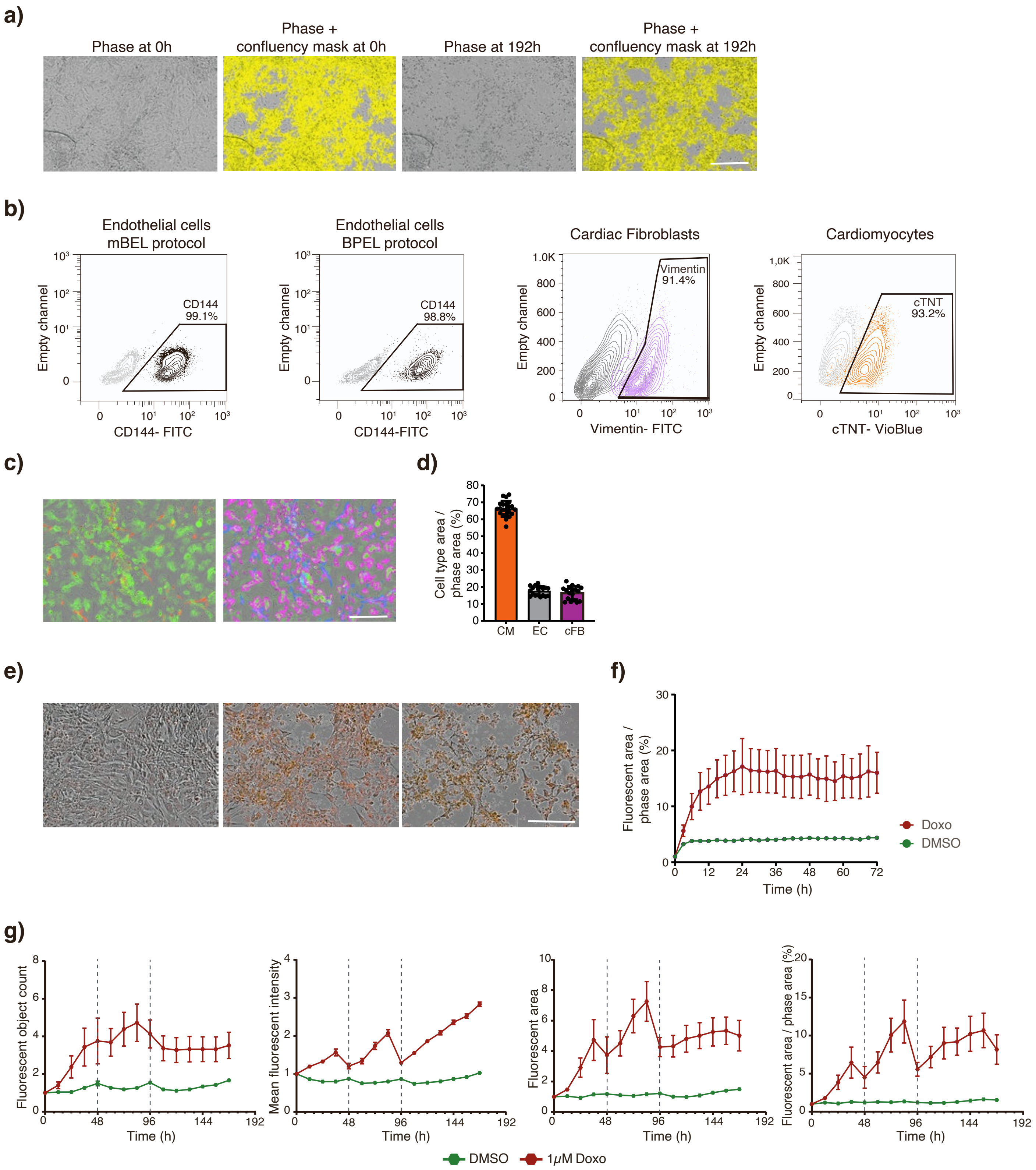

a)
Phase at 0h
Phase + confluency mask at 0h
Phase at 192h
Phase + confluency mask at 192h
b)
Endothelial cells mBEL protocol
Endothelial cells BPEL protocol
Cardiac Fibroblasts
Cardiomyocytes
Empty channel
CD144 99.1%
CD144 98.8%
Vimentin 91.4%
cTNT 93.2%
CD144- FITC
CD144-FITC
Vimentin- FITC
cTNT- VioBlue
c)
d)
Cell type area / phase area (%)
CM
EC
cFB
e)
f)
Fluorescent area / phase area (%)
Time (h)
Doxo
DMSO
g)
Fluorescent object count
Mean fluorescent intensity
Fluorescent area
Fluorescent area / phase area (%)
Time (h)
DMSO
1μM Doxo

Figure S2. Characterization of hiPSC-derived cardiac cell types and limitations of confluency- and fluorescence-based toxicity readouts, related to Figures 1 and 2.

a) Representative phase contrast images of multi-cell type cultures (hiPSC-CMs, -ECs, and -cFBs) with the corresponding phase confluency masks (highlighted in yellow). Masks illustrate the inability of confluency-based metrics to distinguish live from dead cells. Images are shown at baseline (0h) and at 192 h following cumulative 1 μM Doxo treatment, when all cells were visually non-viable. Scale bar, 400 μm.

b) Flow cytometric characterization of hiPSC-derived cell types. Representative plots showing hiPSC-ECs generated using either mBEL- or bPEL-based differentiation protocols expressing CD144, hiPSC-cFBs expressing vimentin and hiPSC-CMs expressing cardiac troponin T (cTnT).

c) Representative merged phase, green and red fluorescence image (*left*) showing the distribution of cell types in a multi-cell type culture (hiPSC-CMs, α-actinin $mEGFP^{+}$; hiPSC-ECs, $mCherry^{+}$; hiPSC-cFBs, non-fluorescent), and the corresponding segmentation masks (*right*) used to quantify each cell type. Scale bar, 400 μm.

d) Proportion of each cell type present in multi-cell type cultures at baseline (0h), based on segmentation of the masks shown in **(c)**. N = 18 wells analyzed.

e) Merged phase and red fluorescence images of hiPSC-CMs labelled with caspase 3/7 dye and treated once with 10 μM Doxo. Images correspond to baseline (0h), 24 h and 72 h after treatment. Scale bar, 200 μm.

f) Time-course quantification of caspase 3/7 fluorescence area relative to phase contrast area (confluency mask) in hiPSC-CMs treated with 10 μM Doxo or DMSO (vehicle control). Data represent mean ± SEM (n = 3 technical replicates).

g) Evaluation of different fluorescence-based metrics for quantifying temporal changes in caspase 3/7 in multi-cell type cultures undergoing cumulative 1 μM Doxo or DMSO treatment. Dotted lines indicate the treatment time points. All values were normalized to baseline (0 h). Data represent mean ± SEM (n = 3 technical replicates).

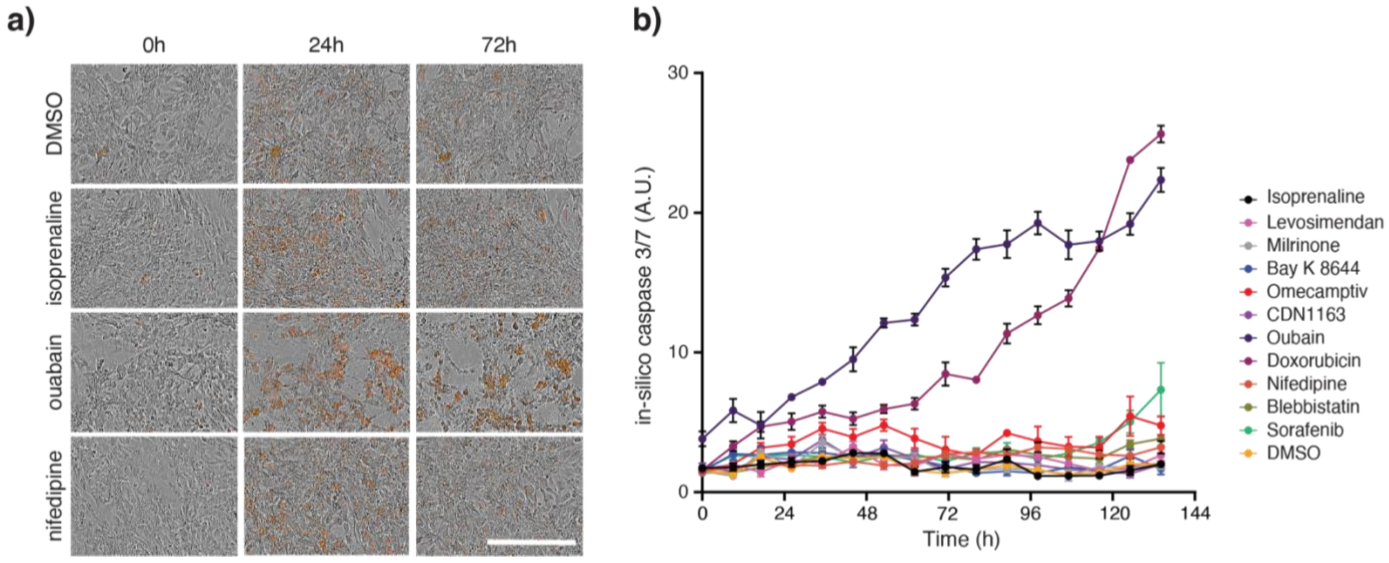

Figure S3. Analysis of caspase 3/7 activity in hiPSC-CMs following treatment with various compounds, related to Figure 3.

a) Merged phase and red fluorescence images of hiPSC-CMs labelled with caspase 3/7 dye and treated once with the indicated compounds. Time points correspond to baseline (0h), 24 h and 72 h after treatment. Scale bar, 400 µm.

b) *In silico* quantification of caspase 3/7 activity over time in hiPSC-CMs treated once with indicated compounds. The treatment concentrations for the compounds are listed in Supplementary Table 2. Data represent mean ± SEM (*n* = 3 biological replicates, each with 3 technical replicates).

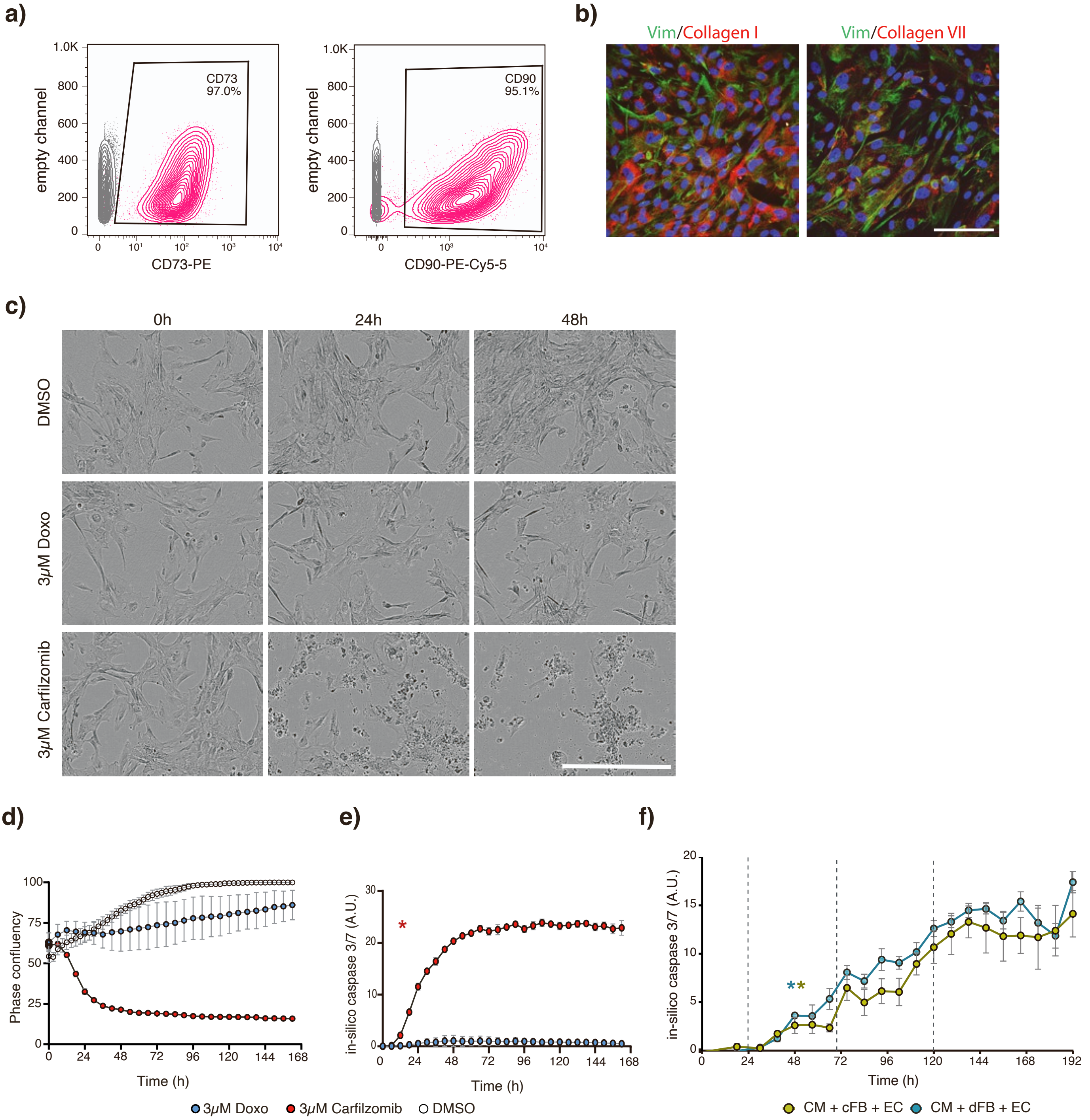

a)
empty channel
CD73
97.0%
CD73-PE
CD90
95.1%
CD90-PE-Cy5-5
b)
Vim/Collagen I
Vim/Collagen VII
c)
0h
24h
48h
DMSO
3μM Doxo
3μM Carfilzomib
d)
Phase confluency
Time (h)
e)
in-silico caspase 3/7 (A.U.)
Time (h)
3μM Doxo
3μM Carfilzomib
DMSO
f)
in-silico caspase 3/7 (A.U.)
Time (h)
CM + cFB + EC
CM + dFB + EC

Figure S4. Characterization of hiPSC-dFBs and their response to cardiotoxic compounds, related to Figure 4.

a) Representative flow cytometry analysis of hiPSC-dFBs expressing the fibroblast surface markers CD73 and CD90.

b) Representative immunofluorescence images showing expression of the fibroblast marker vimentin and extracellular matrix proteins collagen I and collagen VII in hiPSC-dFB**.** Scale bar, 100 µm.

c) Representative phase contrast images of hiPSC-dFBs treated with vehicle control (3 µM DMSO), 3 µM Doxo or 3 µM Carfilzomib. Images were acquired at baseline (0h), and 24 h and 48 h after treatment. Scale bar, 400 µm.

d) Time course quantification of phase confluency area calculated from live cell imaging for the treatment conditions described in **(c)**.

e) *In silico* quantification of caspase 3/7 activity in hiPSC-dFbs for the treatment conditions described in **(c)**, normalized to the vehicle control. The asterisk indicates the first time point at which caspase 3/7 activity was significantly higher than baseline (0 h).

f) *In silico* quantification of caspase 3/7 activity in multi-cell type culture conditions containing either hiPSC-cFBs or hiPSC-dFBs undergoing cumulative 1 µM Doxo treatment (dotted lines). Asterisks indicate the first time point at which caspase 3/7 activity was significantly higher than baseline (0 h).

Statistical significance was determined using two-way repeated measures ANOVA with Sidak's multiple comparison test. Data represent mean ± SEM (*n* = 3 biological replicates, each with 3 technical replicates).

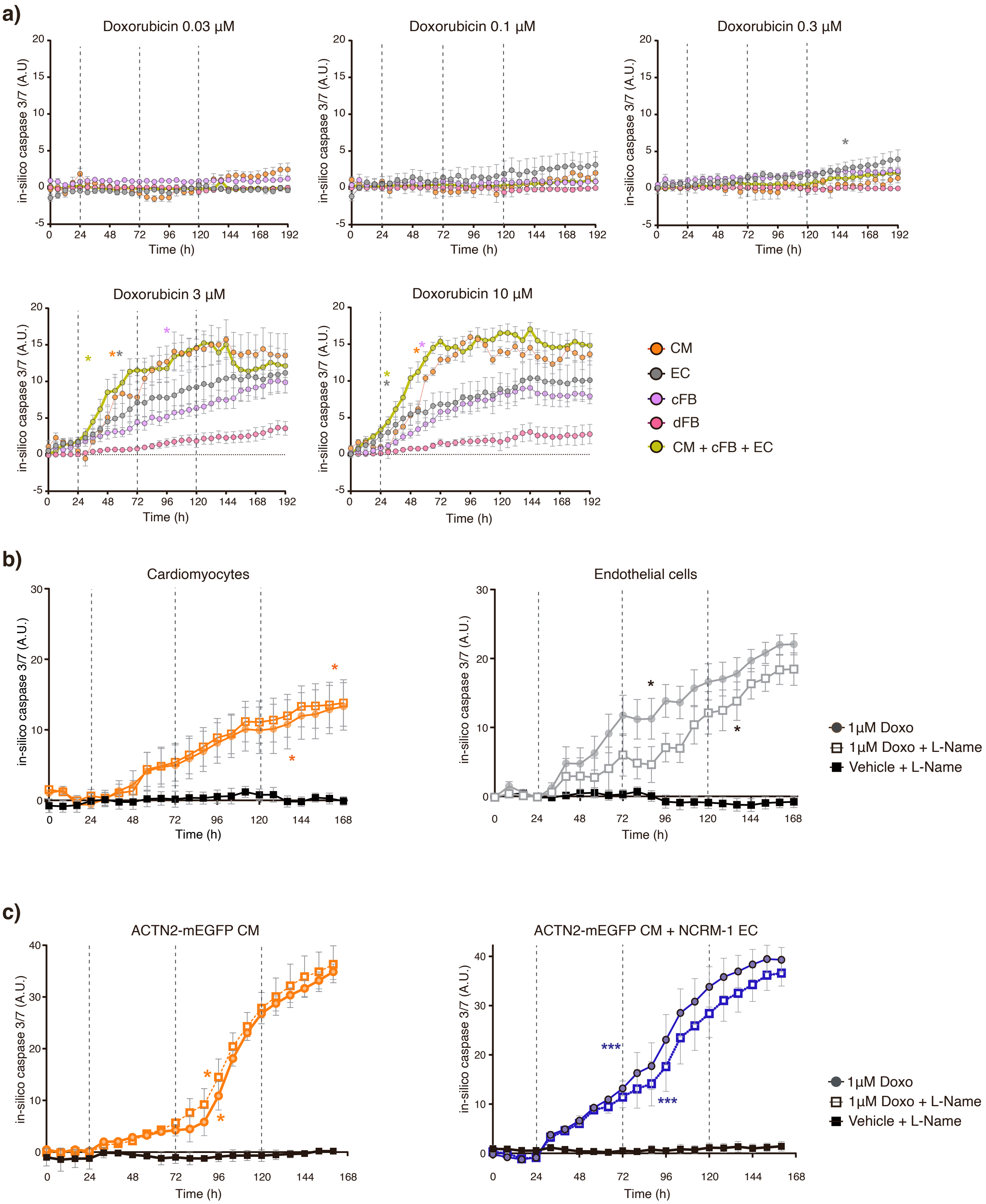

a)
Doxorubicin 0.03 µM
Doxorubicin 0.1 µM
Doxorubicin 0.3 µM
Doxorubicin 3 µM
Doxorubicin 10 µM
in-silico caspase 3/7 (A.U.)
Time (h)
CM
EC
cFB
dFB
CM + cFB + EC
b)
Cardiomyocytes
Endothelial cells
1µM Doxo
1µM Doxo + L-Name
Vehicle + L-Name
c)
ACTN2-mEGFP CM
ACTN2-mEGFP CM + NCRM-1 EC
1µM Doxo
1µM Doxo + L-Name
Vehicle + L-Name

Figure S5. *In silico* quantification of caspase 3/7 activity in isogenic hiPSC-derived cells treated with Doxo and L-Name, related to Figures 4 and 5.

a) *In silico* quantification of caspase 3/7 activity in isogenic monocultures and in multi-cell type culture condition (hiPSC-CMs, -ECs, and -cFBs) treated with various concentrations of Doxo. All treatments (dotted line) followed the cumulative treatment protocol outlined in Fig. 1, except 10 μM Doxo, which was administered as a single exposure.

b) *In silico* quantification of caspase 3/7 activity in hiPSC-CM monocultures (left) and hiPSC-EC monocultures (right) treated with 1 μM Doxo in the presence or absence of 100 μM L-NAME.

c) *In silico* quantification of caspase 3/7 activity in independent sets of hiPSC-differentiated cells: α-actinin-mEGFP hiPSC-CMs (ACTN2-mGFP) in monoculture (left) or co-cultured with NCRM-1 hiPSC-ECs (right) and treated with 1 μM Doxo in the presence or absence of 100 μM L-NAME.

Caspase 3/7 activity was normalized to the corresponding vehicle control for each culture condition. Color-coded asterisks indicate the first time point at which caspase 3/7 activity was significantly higher than baseline (0 h) for each cell type. Statistical significance was determined using two-way repeated measures ANOVA with Sidak's multiple-comparison test. Data represent mean ± SEM (*n* = 3 biological replicates, each with 3 technical replicates).

Table S1. Antibody information, related to Methods.

Antibody	Manufacturer	Identifier	Application
Mouse Anti-Type VII collagen	Sigma Aldrich	C6805; RRID: AB_476860	Immunofluorescence
Mouse Anti-Type I Collagen	Sigma Aldrich	MAB3391; RRID: AB_94839	Immunofluorescence
Rabbit Anti-Vimentin	Thermo Fisher Scientific	PA5-27231; RRID: AB_2544707	Immunofluorescence
Donkey anti-Rabbit IgG (H+L) Highly Cross-Adsorbed Secondary Antibody, Alexa Fluor™ Plus 647	Thermo Fisher Scientific	A32795; RRID: AB_2762835	Immunofluorescence
Donkey anti-Mouse IgG (H+L) Highly Cross-Adsorbed Secondary Antibody, Alexa Fluor™ Plus 555	Thermo Fisher Scientific	A32773; RRID: AB_2762848	Immunofluorescence
PE mouse anti-human CD73	BD Pharmingen	561014; RRID: AB_2033967	Flow cytometry
PE-Cy5 mouse anti-human CD90	BD Pharmingen	561972; RRID: AB_10898004	Flow cytometry
FITC anti-human Vimentin REAfinity	Miltenyi Biotec	130-116-663; RRID: AB_2727645	Flow cytometry
VioBlue anti-human Cardiac Troponin I REAfinity	Miltenyi Biotec	130-120-402; RRID: AB_2783891	Flow cytometry
Alexa 488 anti-human CD144 (VE Cadherin)	Thermo Fisher Scientific	53-1449-42; RRID: AB_10753926	Flow cytometry

Table S2. List of compounds tested, related to Methods.

Compound	Manufacturer	Catalog number	Stock concentration (mM)	Final concentration (μM)
Isoprenaline	Sigma	I5627	10	1
Levosimendan	Merck	141505-33-1	100	0.3
Milrirone	Tocris Bioscience	1504	100	100
Bay K 8644	Tocris Bioscience	1544	100	1
Omecamptiv	Sigma	S2623	1	0.3
CDN1163	Tocris Bioscience	1163	100	10
Oubain	Tocris Bioscience	1076	100	3
Doxorubicin	SelleckChem	S1208	100	0.03 - 10
Nifedipine	Tocris Bioscience	1075	100	0.3
Sorafenib	Tocris Bioscience	6814	20	10
Blebbistatin	Sigma	B0560	10	1
Carfilzomib	SelleckChem	S2853	1	3
DMSO	Sigma	D2650	100	3 - 100
L-NAME	Sigma	N5751	10	100

Supplemental Methods

hPSC culture and differentiation

The medical ethics committee (Leiden University Medical Center) approved the use of hPSC lines in this study (P13.080). All cell lines were routinely tested for mycoplasma using the MycoAlert® Mycoplasma Detecton Kit (Lonza, #LT07-318), and the identity of the cell lines confirmed either by STR analysis (Idexx BioAnalytics) or by visualization of the genetically tagged fluorescent reporters.

The hPSC lines were differentiated into hPSC-CMs using a small molecule protocol [S1]. Briefly, the hPSCs were seeded on Matrigel (Corning)-coated 12 well plates at either 7.5 x 10^4 (HES-3 MESP1^{mcherry}-NKX2.5^{eGFP}), 1.5 x 10^5 (LUMC0020iCTRL-06) or 2.5 x 10^5 (alpha-actinin-2^{mEGFP}) cells/well 24 h (day -1) prior to starting the differentiation. On differentiation day 0, the medium was replaced with mBEL supplemented with 5 µM CHIR99021 (Axon Medchem), followed by mBEL supplemented with 5 µM XAV939 (Tocris Bioscience) and 0.25 µM IWP-L6 (Axon Medchem) on day 2. On day 4, medium was changed to mBEL supplemented with insulin-transferrin-selenium-ethanolamine (ITS-X; Thermo Fisher Scientific). The medium was then replaced every 2-3 days with CM Specification Medium (mBEL minus essential lipids) until the cells were harvested for cryopreservation (differentiation day 14 unless otherwise indicated).

hiPSC-EC differentiation was performed as previously described [S1, S2], with minor modifications. The hiPSCs (LUMC0020iCTRL-06 or NCRM-1) were seeded on Matrigel-coated wells 1 day prior to starting the differentiation. Mesoderm formation was induced in mBEL supplemented with 5 µM CHIR 99021 or B(P)EL medium containing 8 µM CHIR 99021. On differentiation day 3, the medium was replaced with vascular specification medium (either mBEL + ITX-S containing 50 ng/ml VEGF (R&D Systems), or B(P)EL + 50 ng/ml VEGF + 10 µM SB431542 (Tocris Bioscience)), and subsequently refreshed every 2-3 days. The hiPSC-ECs were isolated using either EasyStep™ CD34 Human Cord Blood Isolation Kit II (STEMCELL Technologies) or CD31-Dynabeads™ (Thermo Fisher Scientific) and expanded as previously described for cryopreservation [S1, S2].

The hiPSCs (LUMC0020iCTRL-06) were differentiated into hiPSC-cFB via epicardial progenitor cells [S1], with the modification that the hiPSCs were seeded at 2.0 x 10^4 cells/cm^2 and 5 µM CHIR99021 was used for mesoderm induction (differentiation day 0 to 2). On day 2, the medium was replaced with mBEL supplemented with 5 µM XAV, 1 µM Retinoic Acid (Sigma Aldrich) and 30 ng/ml BMP4 (R&D Systems). From day 4, BMP4 was removed from the culture medium. On differentiation day 9, the epicardial cells were seeded on fibronectin (5 µg/ml bovine plasma; Sigma Aldrich)-coated wells in mBEL supplemented with 10 µM SB431552. When confluent, the epicardial cells were either cryopreserved or further differentiated to cFB by seeding on vitronectin-coated plates and culturing for 6 days in mBEL supplemented with 10 ng/ml FGF2 (R&D Systems). The cFBs were subsequently expanded in Fibroblast Growth Medium 3 (PromoCell), with medium refreshment every 2-3 days until harvested for passaging or cryopreservation.

The hiPSC-dermal fibroblast (hiPSC-dFB) differentiation was essentially as previously described [S3]. Briefly, hiPSCs (LUMC0020iCTRL-06) were aggregated (3.5 x 10^4 cells/well in 100 mL TeSR-E8 + 10 mM Y27632 (Stemgent)) in U-bottom low-attachment 96-well plates (Thermo Fisher Scientific) by centrifugation at 110*g* for 6 min. After 24 h (differentiation day 0), aggregates were collected, washed 3 times with E6 medium (Thermo Fisher Scientific) and transferred to a new U-bottom low-attachment 96-well plate in 100 µL dFB-differentiation medium (E6 medium containing 10 µg/mL TGFb2 (R&D Systems), 0.1 mM L-ascorbic acid (Merck), 1x ITS-A supplement (Thermo Fisher Scientific) and 100 µg/mL Normocin (Invivogen)). After 24 h (differentiation day 1), an additional 50 µL dFB-differentiation medium added to each well. On differentiation day 3, 15-20 aggregates were transferred to each well of a 6-well plate coated with 0.1% gelatin (Sigma Aldrich) and containing DMEM/F12 medium supplemented with 1x GlutaMAX, 20% Fetal Bovine Serum (all Thermo Fisher Scientific) and 0.1 mM L-ascorbic acid. On day 13, cells were harvested using 1x TrypLE Select enzyme (Thermo Fisher Scientific) and replated (dilution 1:3) in dFB-maintenance medium (DMEM/F12 medium + 1x GlutaMAX, 10% Fetal Bovine Serum, 1x non-essential amino acids, 50 U/mL Penicillin, 50 mg/mL Streptomycin, 0.09 mM 2-mercaptoethanol (all Thermo Fisher Scientific), 0.1 mM L-ascorbic acid). The cells were replated at a 1:5 ratio and maintained in dFB-maintenance medium with medium changed every 2 days until wells were confluent for passaging or cryopreservation. Cells were passaged a maximum of 5 times.

All experiments were performed with cryopreserved, differentiated hPSCs. Briefly, thawed hPSC-CMs were cultured on Matrigel-coated wells in mBEL CM Maintenance Medium [S1], with RevitaCell™ Supplement (1:200 dilution) added for the first 24 h to improve recovery. Thawed hiPSC-ECs were cultured on fibronectin-coated wells in mBEL vascular specification medium, while thawed hiPSC-cFB and hiPSC-dFB were cultured in FGM3 or dFB-maintenance medium, respectively, on uncoated wells. The purity of the cryopreserved cell types was evaluated based on criteria previously

described [S1, S3]. Only hPSC-CM batches with >85% cTnT$^+$ cells, hiPSC-cEC batches with >85% CD144 (VE-cadherin)$^+$ cells, hiPSC-cFB batches with >85% Vimentin$^+$ cells, and hPSC-dFB batches with >90% CD73$^+$ and CD90 (Thy-1)$^+$ cells, as determined by flow cytometry, were used.

Between 5-7 days after thawing all cells were harvested using 1x TrypLE Select enzyme, except for the hPSC-CMs which were harvested using 5x TrypLE Select solution. The cells were seeded in 96-well plates. For monocultures, either 6×10^4 hPSC-CMs, 5×10^4 hiPSC-ECs, 1×10^4 hiPSC-cFBs or hiPSC-dFBs were seeded in each well, so that cell confluencies were similar at the start of the experiment. For multi-cell type cultures, a total of 6×10^4 cells plated per well. For cultures containing 3 cell types, the cell composition was 70% hPSC-CMs, 15% hiPSC-ECs, and 15% hiPSC-cFBs or -dFBs, with the cells cultured in mBEL CM Maintenance Medium + 50 ng/ml VEGF and 5 ng/ml FGF2, or a 1:1 mix of mBEL CM Maintenance Medium + 50 ng/ml VEGF and dFB Maintenance Medium, respectively. Cultures containing 2 cell types consisted of 85% hPSC-CMs with either 15% hiPSC-ECs in mBEL CM Maintenance Medium + 50 ng/ml VEGF, or 15% hiPSC-cFBs in mBEL CM Maintenance Medium + 5 ng/ml FGF2.

Flow Cytometry

Single cell suspensions were prepared by washing the cells once with FACS buffer (1× PBS, 0.5% BSA, 2 mM EDTA) and subsequently filtering the suspension. When necessary, cells were fixed and permeabilized using the Fix & Perm Cell Permeabilization Kit (Invitrogen) following the manufacturer's instructions. Cells were incubated with the antibodies listed in **Table S1**. Measurements were acquired using a MACSQuant VYB (Miltenyi Biotec) or LSR II (BD Biosciences) flow cytometer, and data analyzed using FlowJo software (FlowJo, LLC).

Immunofluorescence

Cells were fixed in 1% paraformaldehyde in PBS for 30 min at room temperature (RT). Permeabilization and blocking were performed for 1 h at RT in PBS containing 0.1% Triton X-100 and 4% normal swine serum (Jackson ImmunoResearch, #014-000-121). Cells were incubated with the primary and the appropriate fluorophore-conjugated secondary antibodies (**Table S1**) diluted in PBS for 1 h at RT. Washes were performed with PBS + 0.05% Tween. Nuclei were stained with DAPI (Fisher Scientific, #D3571) and images acquired using a SP5 confocal microscope (Leica).

Cumulative Doxo treatment

For the cumulative treatment protocol, Doxo was diluted to the required concentration in the culture medium appropriate for each cell type. The culture medium on the cells was replaced with 100 µL Doxo-containing medium for 4 h at 37°C, 5% CO_2. Post incubation, the medium was removed and replaced with the standard culture medium. This cycle was repeated 3 times at 48 h intervals for all concentrations of Doxo, except 10 µM which was only incubated with the cells once. DMSO vehicle control concentration (0.1% v/v) matched that of 3 µM Doxo, which was the highest concentration used in the cumulative treatment protocol. Additionally for comparing toxicity, hiPSC-dFBs were treated with 3 µM carfilzomib following an identical protocol and timing.

For NOS-inhibition experiments, L-NAME (100 µM; Sigma #N5751) was included in the treatment medium during each 4 h exposure to Doxo or DMSO vehicle and re-added at every medium change, so that it remained present throughout the entire cumulative treatment period.

Live cell imaging and fluorescence analysis

To detect apoptosis, cells were labelled with either the red or green Incucyte Caspase 3/7 dyes for apoptosis (Sartorius) according to manufacturer's instructions. Briefly, the cells were treated with the Caspase 3/7 reagent at final concentrations of 2.5 µM (red) or 5 µM (green) diluted in the appropriate cell culture medium (100 µL per well), 1 h before Doxo treatment. Initial baseline fluorescence images were obtained prior to drug administration. After drug exposure, the medium was replaced with fresh culture medium containing the Caspase 3/7 dye. This process was repeated at 48 h intervals. Fluorescence imaging using either the red (500 ms acquisition) or green (300 ms acquisition) channels, along with phase contrast imaging, was conducted at regular 3 h intervals.

To analyze phase confluence, eGFP (NKX2.5) expression and caspase 3/7 signal, the Incuycte's integrated software (Incucyte® 2022B Rev2) was used, employing either classic or AI-enhanced confluency modes for phase contrast, and Surface Fit or Top-Hat for fluorescence segmentation depending on the fluorescent channel. Measurements were

normalized to the initial time point (t0) or to phase contrast area for comparative analysis. No image or well was excluded from analysis.

Generation of *in silico* models

Deep neural network (DNN) models were generated to identify hPSC-CMs within a triple culture system and to detect caspase 3/7 activity from phase contrast images. For cardiomyocyte identification, phase contrast images acquired using the Incucyte® system were paired with corresponding fluorescence images depicting NKX2.5 expression, which served as ground truth. Fluorescence images were preprocessed prior to use in model training.

Image segmentation was performed using a U-Net–based architecture [S4] incorporating skip connections to preserve spatial information. The cardiomyocyte model was trained on a dataset comprising 2,500 paired phase contrast and fluorescence images collected over nine consecutive days.

A second DNN model was trained to detect caspase 3/7 activity using a similar workflow. Both models were trained using SmoothL1Loss over 15 epochs with a batch size of 4. The NKX2.5 model utilized a learning rate of 0.00001, while the Caspase model employed 0.0001. The performance of each model was evaluated by comparing the accuracy of the predicted labelling to actual labelled images (NKX2.5: 7200 analyzed images; Caspase: 8537 analyzed images) not used in the training of the model.

in silico image analysis and data processing

Archives of the phase contrast images, which included imaging data collected throughout the experiment, along with detailed plate map of conditions and preliminary analyses conducted using the Incucyte® system's integrated software (basic well confluency), were exported from the Incucyte®. No image or well was excluded from the analysis.

This data was subsequently analyzed with the caspase 3/7 and NKX2.5 DNN tools (Sartorius) using Python programming language and the Pytorch framework for deep learning. *In silico* caspase 3/7 output data was further processed in R (version 4.3.2). For each time point and cell type, DMSO vehicle control values were averaged across 3 technical replicate wells and subtracted from individual caspase 3/7 measurements. The resulting values were normalized to the initial time point (0 h).

The *in silico* NKX2.5^{+} values were normalized by dividing each time point by the corresponding initial value (0 h). Normalization and correction procedures were performed separately for each biological replicate.

Supplemental References

[S1] Campostrini, G., Meraviglia, V., Giacomelli, E., van Helden, R.W.J., Yiangou, L., Davis, R.P., Bellin, M., Orlova, V.V., and Mummery, C.L. (2021). Generation, functional analysis and applications of isogenic three-dimensional self-aggregating cardiac microtissues from human pluripotent stem cells. Nat. Protoc. *16*, 2213-2256. 10.1038/s41596-021-00497-2.

[S2] Orlova, V.V., van den Hil, F.E., Petrus-Reurer, S., Drabsch, Y., Ten Dijke, P., and Mummery, C.L. (2014). Generation, expansion and functional analysis of endothelial cells and pericytes derived from human pluripotent stem cells. Nat Protoc *9*, 1514-1531. 10.1038/nprot.2014.102.

[S3] Itoh, M., Umegaki-Arao, N., Guo, Z., Liu, L., Higgins, C.A., and Christiano, A.M. (2013). Generation of 3D skin equivalents fully reconstituted from human induced pluripotent stem cells (iPSCs). PLoS One *8*, e77673. 10.1371/journal.pone.0077673.

[S4] Falk, T., Mai, D., Bensch, R., Çiçek, Ö., Abdulkadir, A., Marrakchi, Y., Böhm, A., Deubner, J., Jäckel, Z., and Seiwald, K. (2019). U-Net: deep learning for cell counting, detection, and morphometry. Nat. Methods *16*, 67-70. 10.1038/s41592-018-0261-2.
