# Supplementary material for: A simplified co-culture reveals altered cardiotoxic responses to doxorubicin in hPSC-derived cardiomyocytes in the presence of endothelial cells
Source: Stem Cell Reports. 2026 Feb 12;21(3):102816. doi: 10.1016/j.stemcr.2026.102816 (PMC12985372; doi:10.1016/j.stemcr.2026.102816)
Supplement: Document S2. Article plus supplemental information [file mmc2.pdf]

STEM CELL REPORTS
Article
OPEN ACCESS

A simplified co-culture reveals altered cardiotoxic responses to doxorubicin in hPSC-derived cardiomyocytes in the presence of endothelial cells

Marcella Brescia,[1] James Gallant,[1,4] Andrea Chatrian,[2] Paul Keselman,[3] Elsa Sörman Paulsson,[2,9] Mervyn P.H. Mol,[1] Rickard Sjögren,[2,9] Karine Raymond,[1,4,5] Valeria Orlova,[1,4] Kalpana Barnes,[6] Richard Wales,[7] Jonas Austerjost,[8] Michael W. Olszowy,[3] Christine L. Mummery,[1,4] Berend J. van Meer,[1,10,*] and Richard P. Davis[1,4,11,*]
[1]Department of Anatomy and Embryology, Leiden University Medical Center, Leiden, the Netherlands
[2]Sartorius Stedim Data Analytics Ab, Corporate Research, Umeå, Sweden
[3]Sartorius Stedim North America Inc., Corporate Research, Bohemia, NY, USA
[4]The Novo Nordisk Foundation Center for Stem Cell Medicine, reNEW, Leiden University Medical Center, Leiden, the Netherlands
[5]University of Grenoble Alpes, CEA, INSERM, IRIG, UA13 BGE, Biomics, Grenoble, France
[6]Essen Bioscience Ltd., Product Development, Royston, UK
[7]The Automation Partnership (Cambridge) Ltd., Corporate Research, Royston, UK
[8]Sartorius Stedim Biotech GmbH, Corporate Research, Göttingen, Germany
[9]Present address: SynGen AI Technologies AB, Umeå, Sweden
[10]Present address: Sync Biosystems, Leiden, The Netherlands
[11]lead contact
*Correspondence: berend.van.meer@demcon.com (B.J.v.M.), r.p.davis@lumc.nl (R.P.D.)
https://doi.org/10.1016/j.stemcr.2026.102816

SUMMARY

Cardiotoxicity is a significant challenge in cancer therapies, particularly with doxorubicin, a widely used anthracycline. More predictive *in vitro* models are needed to understand doxorubicin-induced cardiac damage and patient-specific responses. Here, human pluripotent stem cell (hPSC)-derived cardiomyocytes (hPSC-CMs), cardiac fibroblasts (hPSC-cFBs), and endothelial cells (hPSC-ECs) were cultured in mono- or multi-cell-type formats and repeatedly treated with doxorubicin to mimic cumulative clinical exposure. A machine learning-based tool enabled continuous quantification of the early toxicity marker caspase-3/7 and accurately identified hPSC-CMs within mixed cultures. Notably, hPSC-ECs were more sensitive to doxorubicin than hPSC-CMs or hPSC-cFBs, with nitric oxide signaling contributing to the elevated cardiomyocyte toxicity observed in co-culture. These results question the conventional *in vitro* focus on cardiomyocytes regarding drug-induced cardiac damage, highlighting the interplay among different cardiac cell types in mediating the toxic effects of doxorubicin. Furthermore, the work demonstrates the potential of AI-based tools to provide scalable strategies for assessing drug-induced cardiotoxicity.

INTRODUCTION

Predicting patient-specific drug responses and adverse reactions remains a significant challenge in healthcare, particularly in the context of cardiotoxicity. This is most notable with chemotherapeutics, which can have well-known life-threatening side effects with late heart failure evident in up to 10% of patients (van der Pal et al., 2012). The variability in drug efficacy and safety, even among patients with identical diagnoses, complicates the prediction of such adverse effects (Schwach et al., 2024). The heart is one of several organs for which regulatory authorities mandate comprehensive cardiotoxicity assessments throughout the drug development process (Kettenhofen and Bohlen, 2008). Conventionally, these assays have predominantly relied on *ex vivo* assays and *in vivo* studies in animals. However, the physiological and anatomical differences between humans and most animal models often limit the translatability of such studies, contributing to high failure rates (~90%) of drug candidates in clinical trials due to unforeseen toxicities and lack of efficacy (Mak et al., 2014; Zaragoza et al., 2011). Contemporary regulatory initiatives, exemplified by the FDA Modernization Act 2.0, are driving a paradigm shift away from reliance on animal models toward the adoption of cell-based assays and advanced data analysis methodologies (Zushin et al., 2023). Human pluripotent stem cells (hPSCs) are emerging as a promising alternative, potentially offering more predictive models for evaluating drug-induced cardiotoxic effects and reducing reliance on animals (Saleem et al., 2020). Moreover, these cells are particularly valuable for personalized screening and improving understanding of why individual patients respond differently to cardiotoxic compounds (de Korte et al., 2020).

Doxorubicin (Doxo), a widely used anthracycline, exemplifies the delicate balance between therapeutic efficacy and cardiotoxic risk. Despite its proven effectiveness in treating a variety of cancers, including acute leukemia, lymphomas, and various solid tumors in both adults and children, it is also associated with significant adverse effects, most notably cardiotoxicity (Linders et al., 2024).

© 2026 The Author(s).
Published by Elsevier Inc. on behalf of International Society for Stem Cell Research.
This is an open access article under the CC BY license (http://creativecommons.org/licenses/by/4.0/).

This cardiotoxic effect, characterized by a spectrum of cardiac dysfunctions such as congestive heart failure, arrhythmias, and reduced left ventricular ejection fraction (Csapo and Lazar, 2014), manifests in a cumulative and dose-dependent manner and severely limits its repeated or long-term clinical use. The mechanisms involved are complex and not fully understood, but include mitochondrial dysfunction, calcium overload, DNA damage, and apoptosis following caspase activation (Karabulut et al., 2021; Michihiko et al., 2006; Rawat et al., 2021).

hPSC-derived cardiomyocytes (hPSC-CMs) have become a mainstream tool for cardiotoxicity testing by both the pharmaceutical industry and academia, owing to their scalability and suitability for high-throughput safety assessments (Raniga et al., 2024; Stebbeds et al., 2023). However, despite their widespread adoption, these models still present limitations in accurately predicting clinical outcomes. Increasing their complexity by incorporating additional hPSC-derived cardiac cell types such as endothelial cells (ECs) and cardiac fibroblasts (cFBs), has been shown to improve their physiological relevance and consequently, their clinical translatability (Raniga et al., 2024). This improvement is particularly evident in 3D cultures, for example, microtissues or engineered heart tissues (EHTs), which promote not only the maturation of hPSC-CMs but also the predictiveness of drug responses (Giacomelli et al., 2020; Saleem et al., 2020), including the effects of Doxo on contraction dynamics (Qiao et al., 2020; Schwach et al., 2024).

However, it currently remains challenging to investigate cell type-specific responses in three-dimensional (3D) models, particularly in real time. In this context, we investigated the potential of two-dimensional (2D) multi-cell-type cultures to offer a more predictive model than 2D hPSC-CM monocultures, while remaining more accessible than 3D cardiac models for both drug exposure and imaging. To analyze the dynamics of cardiotoxicity over prolonged and repeated exposures to Doxo and quantitatively compare the effects between different cell types, we applied a machine learning (ML)-based analysis tool to assess caspase-3/7 activity as a marker of early toxicity. This *in silico* tool could also identify hPSC-CMs in multi-cell-type cultures, allowing us to track cardiomyocyte apoptosis in different cellular environments over time. Our findings revealed that the presence of specific cell types, notably hPSC-ECs, influenced the sensitivity of hPSC-CMs to Doxo and may be a key driver of cardiomyocyte toxicity. This study not only sheds light on Doxo-induced cardiotoxic mechanisms but also underscores the potential of AI tools to advance high-throughput analysis and personalized drug screening, paving the way for safer therapeutic interventions.

RESULTS

hPSC-CMs co-cultured with other cardiac cell types appear more sensitive to Doxo

To emulate the *in vivo* pharmacodynamics of Doxo (Barpe et al., 2010; Pang et al., 2013), an *in vitro* treatment protocol was used in which the hPSC-derived cells were exposed to Doxo for 4-h intervals every 48 h (Figure 1A). Because the free diffusible Doxo concentration *in vivo* varies between 20 nM and 2 μM (Greene et al., 1983), we examined a range of concentrations (0.01–10 μM), with representative phase contrast images and analysis presented in Figure S1. We selected 1 μM Doxo as a proxy for the cardiotoxic effect seen *in vivo* since this concentration induced cumulative toxicity without immediate cell death in hiPSC-CMs.

When hiPSC-CMs were treated in co-culture with hiPSC-cFBs and hiPSC-ECs, Doxo induced toxicity more rapidly than in monocultures of hiPSC-CMs. In phase contrast images, cell death was immediately evident after a single 1 μM Doxo treatment in triple cultures, while monocultures of hiPSC-CMs required 3 cycles for visible cell death (Figure 1B). Through frequent imaging, morphological changes indicating of apoptosis became evident, including cytoplasmic shrinkage, cell shape changes, and cell detachment. However, when attempting to quantify the extent of toxicity, conventional phase microscopy was unable to distinguish live from dead cells (Figure S2A) due to cellular debris and clumping, which affected the ability to apply size- or eccentricity-based threshold filters. This resulted in discrepancies with, for example, confluency measurements indicating only 20% loss in the co-culture condition at 96 h (Figure 1C), despite most cells appearing dead (Figure 1B). Nevertheless, we could distinguish differences in the toxicity dynamics between triple cultures and hiPSC-CM monocultures, with triple cultures exhibiting a more rapid onset of cell death following Doxo treatment (Figure 1C). The composition of the triple culture (70% hPSC-CMs, 15% hiPSC-ECs, and 15% hiPSC-cFBs) prior to Doxo treatment was confirmed (Figures S2B–S2D).

From video recordings of the hiPSC-cardiac cultures acquired during their treatment, differences in the beat rate of the hiPSC-CMs between the mono- and triple culture conditions were also observed (Figure 1D). Contraction traces were analyzed using CardioMotion software 24 h after the first and second treatments with either 1 or 3 μM Doxo (Stebbeds et al., 2023). Contractility was not assessed after the third cycle of Doxo treatment due to significant cell death. Although contraction amplitudes did not initially differ significantly between culture types, co-cultures had notably lower amplitudes after the second Doxo treatment (Figure 1E). Additionally, analysis of contraction duration parameters, specifically relaxation time and time to peak (Figures 1F and 1G), revealed

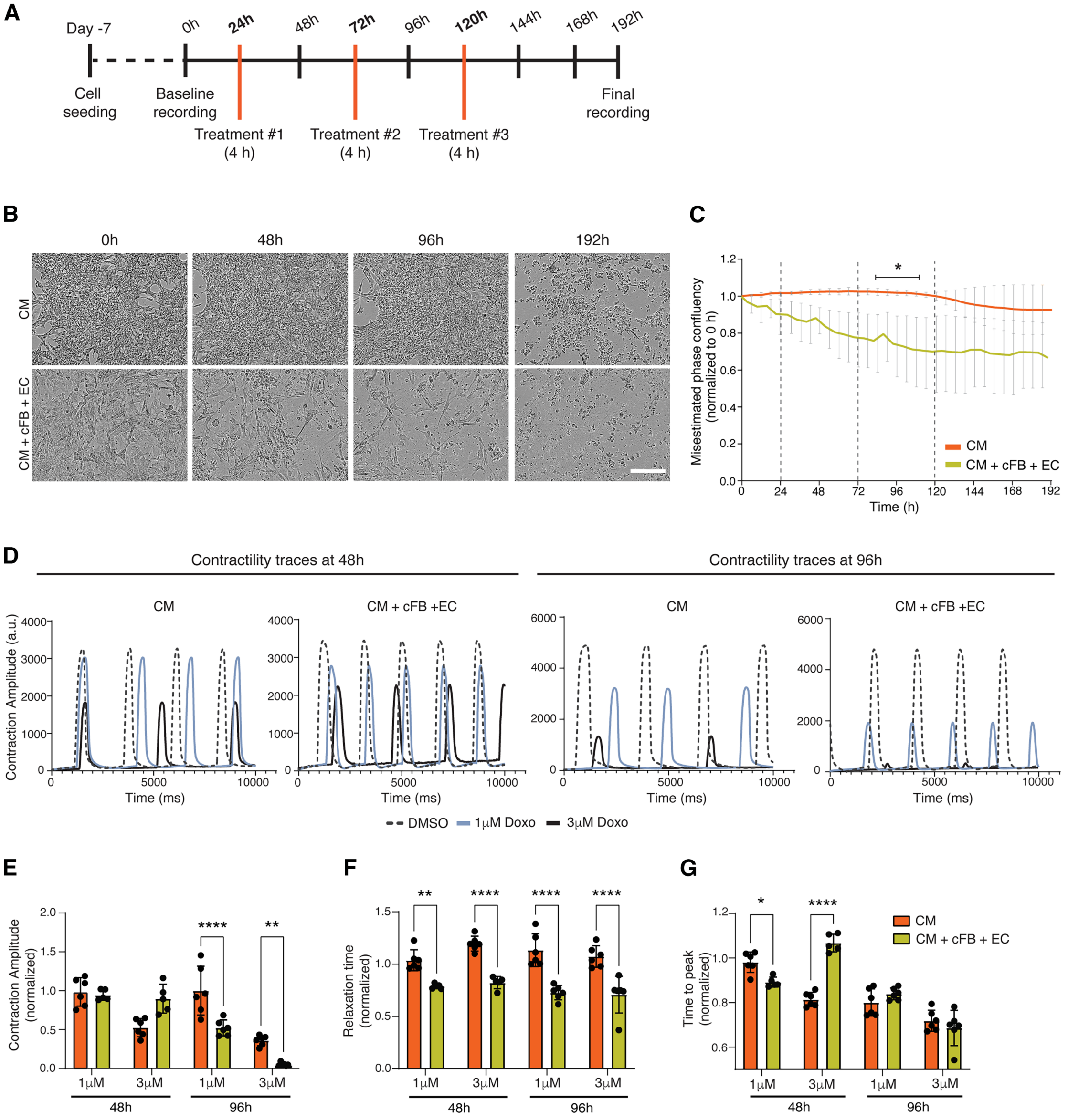

Figure 1. Cumulative Doxo treatment effect on hPSC-derived cardiac cell cultures

(A) Schematic of treatment protocol timeline, with orange lines marking the time points of 4-h Doxo application to the cells.

(B) Representative phase contrast images of monoculture hiPSC-CMs (top) or co-cultured with hiPSC-cFBs and -ECs (bottom) treated with 1 μM Doxo. Images were acquired at time points corresponding to baseline (0 h), 24 h after treatments 1 and 2 (48 and 96 h, respectively), and the final time point (192 h). Scale bar, 100 μm.

(C) Quantification of phase confluency area calculated from live cell imaging, and normalized to baseline (0 h), for conditions shown in (B), highlighting challenges in accurately determining confluency. Dotted lines indicate treatment time points with 1 μM Doxo. The asterisk and black bar indicate the time points where there were statistically significant differences between the monoculture and the multi-cell-type culture setups; *$p < 0.05$.

(D) Representative contraction traces of hiPSC-CMs in both monoculture and multi-cell-type conditions at 48 and 96 h (24 h after treatments 1 and 2, respectively). Treatments were either DMSO (vehicle control) or 1 or 3 μM Doxo.

(E–G) Graphs comparing contraction amplitude (E), relaxation time (F), and time to peak (G) of hiPSC-CMs in either monoculture or multi-cell-type culture conditions at the indicated time points. Each was normalized to their respective vehicle control. Statistical significance was determined by two-way ANOVA analysis. Analysis is based on 3 biological replicates, each with 3 technical replicates, with error bars representing SEM; *$p < 0.05$, **$p < 0.01$, ****$p < 0.0001$. See also Figures S1 and S2.

significantly shorter relaxation times in hiPSC-CMs within the triple cultures after 1 round of Doxo treatment, suggesting more pronounced cardiotoxicity in the presence of ECs and cFBs.

To visualize the effect of Doxo on the hPSC-CMs directly, we used an NKX2.5-eGFP human embryonic stem cell (hESC) reporter line in which cells differentiating to cardiomyocytes express eGFP (Elliott et al., 2011). This confirmed the increased sensitivity of triple cultures to Doxo compared to monocultures, with overall cell confluency significantly decreasing after the first Doxo treatment (Figures 2A and 2B). The decrease in GFP area (corresponding to NKX2.5$^+$ hESC-CMs) paralleled the decrease in phase confluency, indicating that the hESC-CMs were also dying sooner in response to Doxo when co-cultured with the other cardiac cell types (Figure 2C). However, again here it was challenging to accurately quantify cell death with conventional phase microscopy analysis. For example, in cultures exposed to multiple treatment rounds of 1 μM Doxo, the monocultures appeared to proliferate due to increased cell spreading before a decrease in confluency after 80 h was observed (Figures 2B and 2C). Furthermore, while visual inspection confirmed 100% cell death at the last time point, quantitative analysis based on confluency only indicated an ~60% reduction in both phase confluency and GFP area.

We investigated then whether we could quantify caspase activation as a measure of Doxo-mediated apoptosis using a fluorescence assay. Although this was possible when hPSC-CMs were treated with a single high dose (10 μM) of Doxo (Figures S2E and S2F), the utility of the assay was limited in the cumulative treatment protocol with abrupt peaks in the fluorescence signal observed in all analyses performed (Figure S2G). This suboptimal fluorescence quantification was likely due to artifacts introduced with the re-addition of the dye after each medium replacement and the loss of labeled cells with washes.

Our findings thus indicated that hPSC-CMs in multi-cell-type cultures of cFBs and ECs were more sensitive to Doxo-induced toxicity than hPSC-CMs cultured alone. However, due to the complexity of the treatment protocol, accurate quantification was not possible using standard image processing assays indicating the need for alternative analytical techniques, which we next sought to address.

In silico prediction software can accurately detect and quantify hPSC-CMs and caspase activation

We investigated whether the deep neural network (DNN) tools could facilitate and improve the quantification of toxicity evident in the phase images, as well as identify specific cell types. When comparing fluorescence images of multi-cell-type cultures that contained NKX2.5-eGFP$^+$ hESC-CMs and hiPSC-cFBs and -ECs, and exposed to different treatments, with images in which the NKX2.5-expressing cells were determined using the DNN, we observed a mean accuracy of 74.4% across all predictions (Figures 3A and 3B). Further analysis confirmed that *in silico* NKX2.5 predictions closely matched actual NKX2.5-eGFP measurements in multi-cell-type cultures treated with either a lethal concentration of Doxo (10 μM) or DMSO (Figure 3C).

Additionally, we applied a DNN to identify cells undergoing caspase-3/7-mediated apoptosis from phase contrast images. Also here, the hPSC-derived cells that were predicted by the DNN analysis to express caspase-3/7 closely matched the actual labeling, with a mean accuracy of 77.3% across the various cell types, time points, and treatments analyzed (Figures 3D and 3E). Quantification using the DNN also appeared more reliable, detecting high levels of caspase activity over the entire duration of the experiment, compared to the caspase-3/7 dye in which the signal intensity waned over time, particularly after medium changes (Figure 3F).

We then reanalyzed the phase contrast and fluorescence imaging data collected of the mono- and triple cultures of the NKX2.5^{eGFP} hESC-CMs treated with multiple rounds of 1 μM Doxo (Figure 2) using the DNN tools. Quantification better reflected the visual observation, namely, that triple cultures were more sensitive to Doxo-mediated apoptosis (Figure 3G) and that the hPSC-CM numbers declined more quickly in these wells than in monocultures (Figure 3H). We also analyzed data collected from hPSC-CMs exposed to both toxic and non-toxic cardiac-relevant drugs using the DNN caspase-3/7 software (Figure S3). As expected, caspase-3/7 activity was only predicted in the cultures treated with either Doxo or ouabain, a Na^+/K^+-ATPase inhibitor also known for its cardiotoxicity (Sapia et al., 2010). Other compounds, such as isoprenaline and nifedipine, as well as DMSO, were not predicted to induce caspase-3/7 activity, consistent with observations in cultures labeled with the caspase-3/7 dye (Figure S3A).

Differential Doxo toxicity responses between isogenic cardiac and non-cardiac cell types

To investigate whether the higher sensitivity of cells in the triple cultures to Doxo might be due to differential sensitivity of one cell type, we separately exposed each cell type to the cumulative Doxo treatment. We also included hiPSC-derived dermal fibroblasts (dFBs), differentiated from the same hiPSC line, as a non-cardiac cell type for comparison (Figure S4A). Phase contrast images clearly indicated that ECs were affected after a single round of exposure to 1 μM Doxo, while cFBs showed delayed cumulative effects (Figure 4A). Interestingly, the hiPSC-dFBs were not only resistant to the cumulative Doxo treatment but also continued to proliferate.

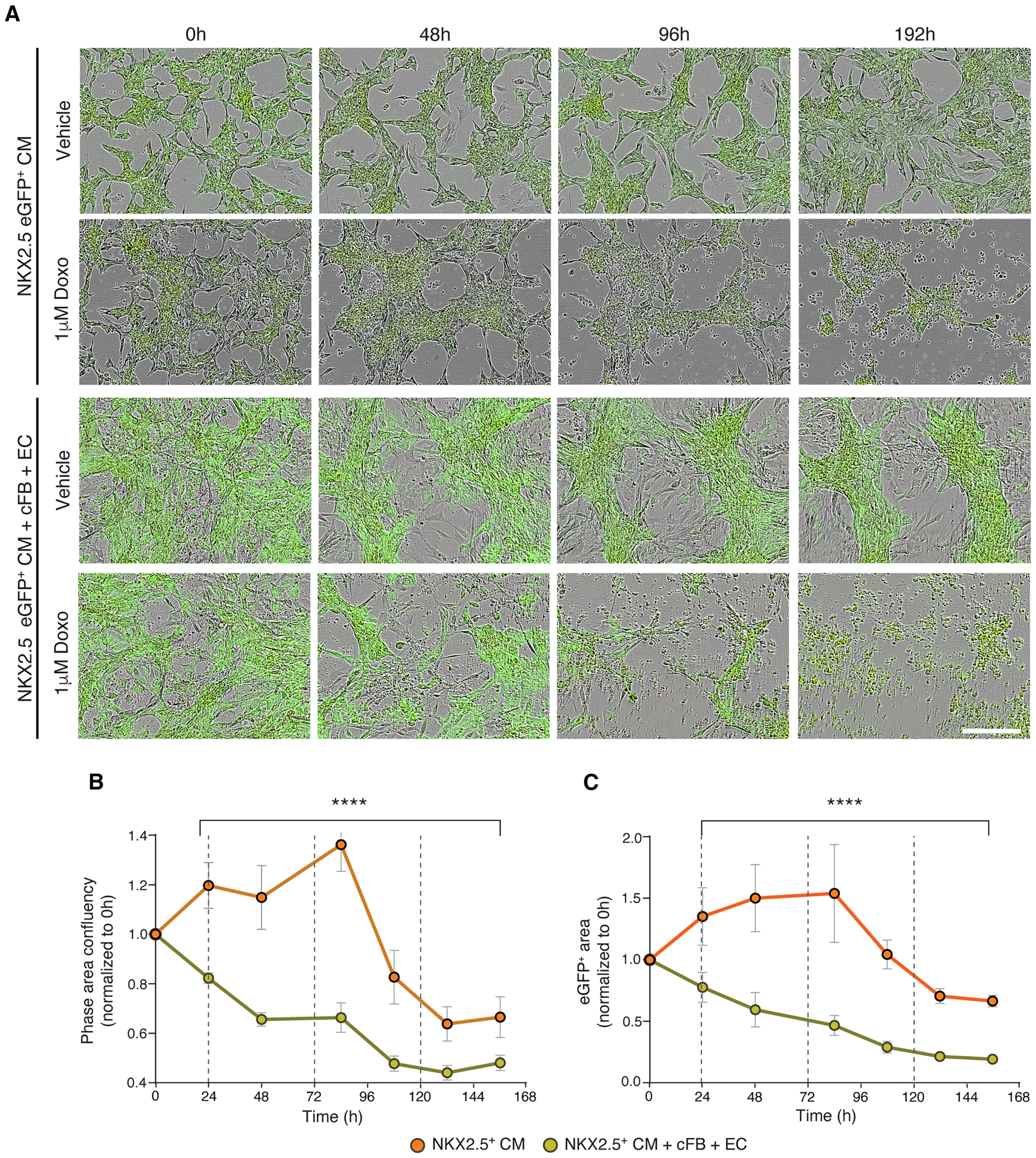

Figure 2. Cumulative Doxo treatment effect on hESC-CMs in mono- or tri-cellular cultures

(A) Representative merged phase and green fluorescence images of NKX2.5-eGFP$^+$ hESC-CMs in monoculture or co-cultured with hiPSC-cFBs and -ECs, treated with either 1 μM Doxo or vehicle control (DMSO) following the cumulative treatment protocol. Images were acquired at time points corresponding to baseline (0 h), 24 h after treatments 1 and 2 (48 and 96 h, respectively), and the final time point (192 h). Scale bar, 100 μm.

(B and C) Quantification of phase (B) and green (NKX2.5$^+$ hESC-CMs, C) confluency areas calculated from live cell imaging and normalized to baseline (0 h), for cell culture conditions outlined in (A) treated with 1 μM Doxo. Dotted lines indicate the treatment time points. Statistical analysis between cell culture conditions employed two-way ANOVA analysis for each time point. Analysis is based on 3 biological replicates, each with 3 technical replicates, with error bars representing SEM; ****p < 0.0001. See also Figure S2.

Quantitative evaluation using the DNN caspase-3/7 tool supported the above observations, as well as the differential response observed in Figure 1B with the hiPSC-CM mono-cultures and triple cultures from this cell line (Figure 4B). Distinct temporal toxicity profiles were detected for each cell type, indicating variable sensitivities to Doxo. Among

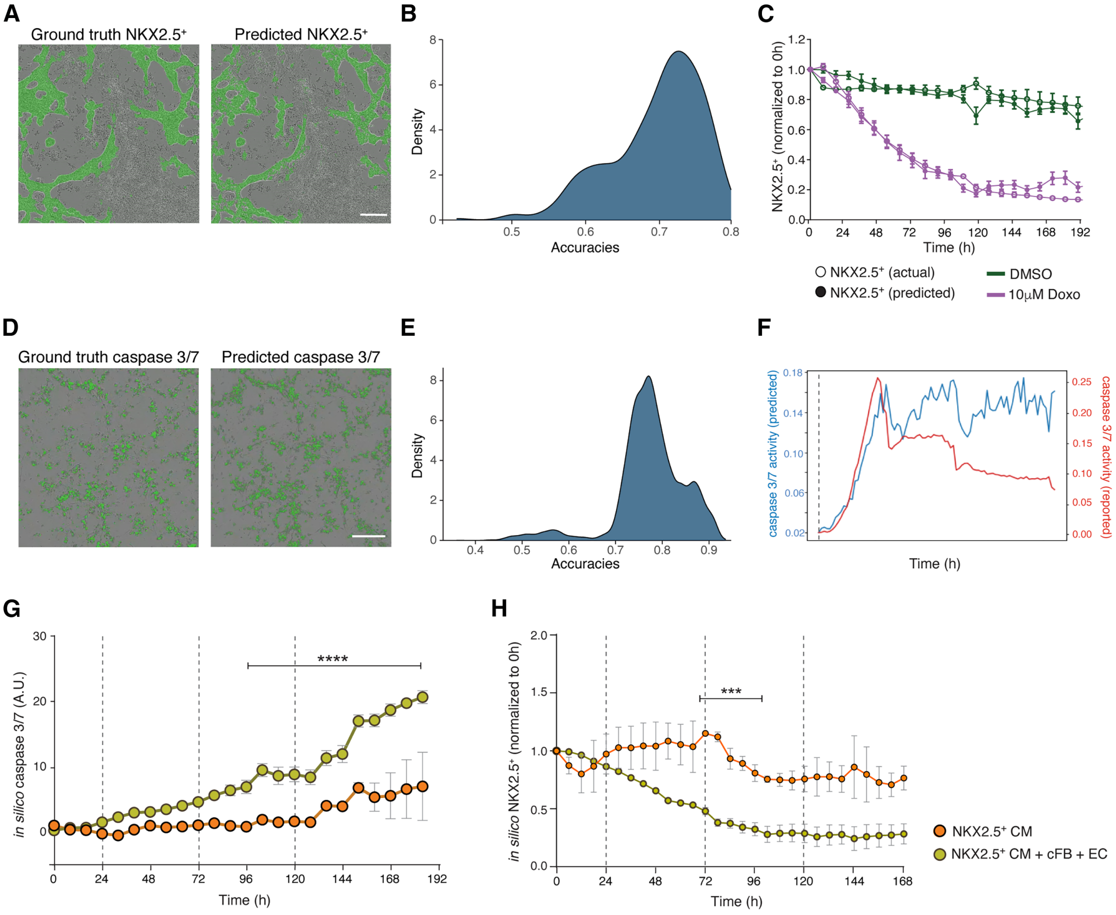

Figure 3. Validating the DNN tools for cardiomyocyte and caspase activity prediction

(A) Matched images comparing actual NKX2.5-eGFP+ hESC-CMs (ground truth) merged with phase contrast to the corresponding *in silico*-predicted NKX2.5-eGFP+ cardiomyocytes derived from phase contrast imaging. Scale bar, 200 μm.

(B) Analysis evaluating the distribution of the accuracy of predicted *in silico* masks against a total of 7,200 actual NKX2.5-eGFP+ images.

(C) Graph depicting the overlap between *in silico* NKX2.5+ predictions and actual NKX2.5+ measurements over time, normalized to baseline (0 h). Lines represent control cells treated with DMSO (green) and those exposed to single 10 μM Doxo treatment (purple).

(D) Matched images comparing actual caspase-3/7 expression (ground truth) merged with phase contrast to the corresponding *in silico*-predicted caspase-3/7 expression derived from phase contrast imaging. Scale bar, 200 μm.

(E) Analysis evaluating the distribution of the accuracy of predicted *in silico* masks against known caspase-3/7 fluorescent dye values from a total of 8,537 images.

(F) Time-course comparison of caspase-3/7 activity, contrasting *in silico* predictions with quantification obtained for caspase-3/7 dye-labeled cells treated with 10 μM Doxo.

(G and H) *In silico* quantification of caspase-3/7 activity (G) and NKX2.5-eGFP+ hESC-CMs, normalized to baseline (0 h) (H), in monoculture versus multi-cell-type culture conditions undergoing cumulative treatment with 1 μM Doxo (dotted lines). Asterisks and black bars indicate the time points where there are statistically significant differences between the monoculture and the multi-cell-type culture setups. Statistical analysis was performed with two-way ANOVA for each time point. Analysis is based on 3 biological replicates, each with 3 technical replicates, with error bars representing SEM; ***$p < 0.001$, ****$p < 0.0001$. See also Figure S3.

the individual cell types, hiPSC-ECs were the most sensitive, displaying significant *in silico* caspase-3/7 activity within 75 h of the initial Doxo treatment. In contrast, both cFBs and dFBs showed overall less caspase activation, with dFBs expressing almost no caspase-3/7, reflecting observations in the phase contrast images. To determine if the toxicity resistance of the hiPSC-dFBs was Doxo specific, we treated these cells with carfilzomib, a proteasome inhibitor not only used clinically to treat multiple myeloma but also known to cause broad tissue toxicity (Georgiopoulos et al., 2023). In contrast to their Doxo response, dFBs were sensitive to carfilzomib, showing significant cell death and

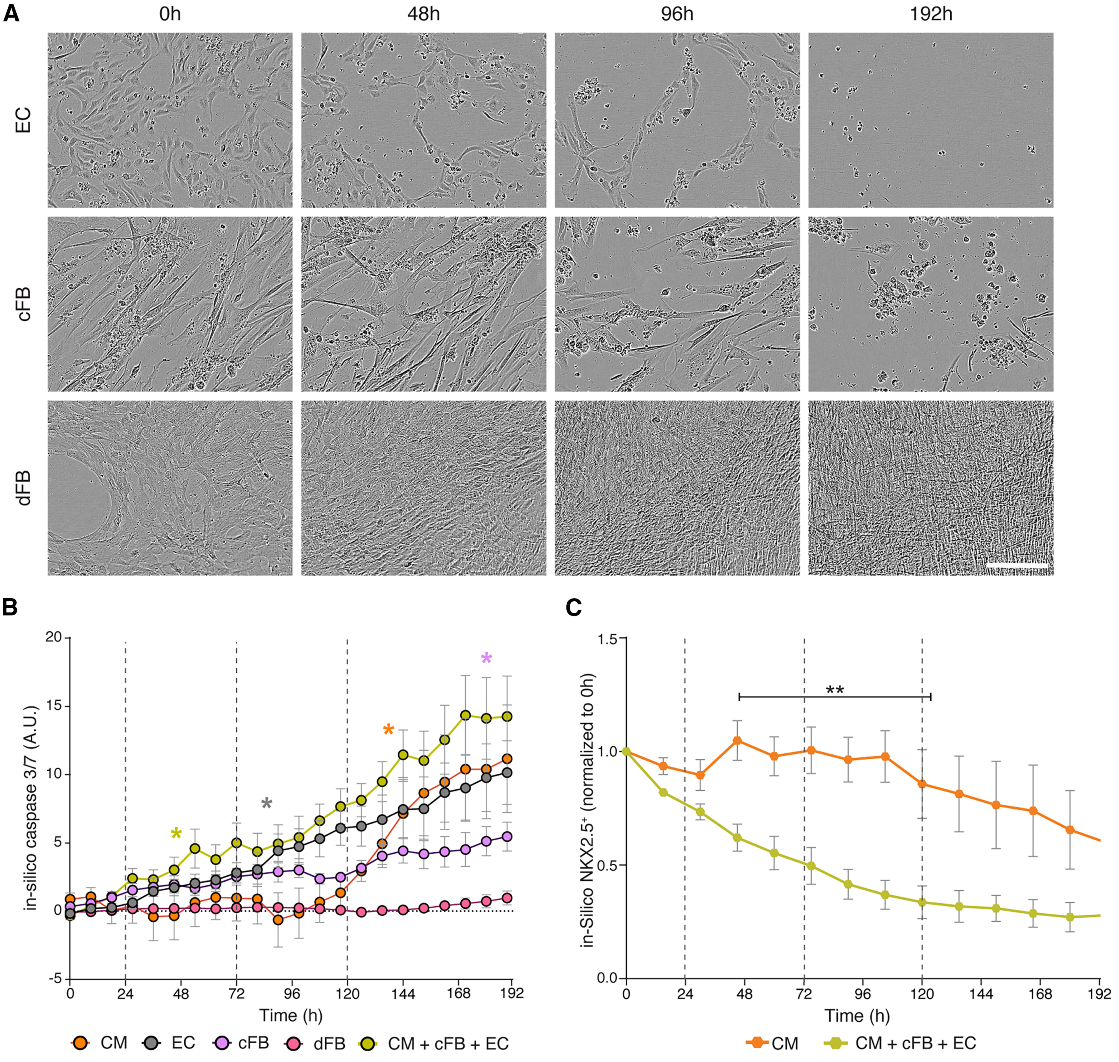

Figure 4. Differential Doxo sensitivity of individual isogenic hiPSC-derived cell types

(A) Representative phase contrast images of monocultures of hiPSC-ECs (top), -cFBs (middle), and -dFBs (bottom) undergoing cumulative treatment with 1 μM Doxo. Images were acquired at time points corresponding to baseline (0 h), 24 h after treatments 1 and 2 (48 and 96 h, respectively), and the final time point (192 h). Scale bar, 100 μm.

(B) *In silico* quantification of caspase-3/7 activity for all isogenic monocultures, as well as the tri-cellular culture condition, undergoing cumulative treatment with 1 μM Doxo (dotted lines). Color-coded asterisks indicate the initial time point at which caspase-3/7 activity is significantly higher ($p < 0.05$) than baseline (0 h) for each cell type. Statistical analysis was performed using a two-way repeated-measures ANOVA with Geisser-Greenhouse correction. Analysis is based on 3 biological replicates, each with 3 technical replicates, with error bars representing SEM; $^{*}p < 0.05$.

(C) *In silico* quantification of $NKX2.5^{+}$ cells in monoculture hiPSC-CMs versus tri-cell-type culture conditions. The asterisk and black bar indicate the time points where there are statistically significant differences between the 2 cultures ($^{**}p < 0.01$). Statistical analysis was performed with two-way ANOVA. Analysis is based on 3 biological replicates, each with 3 technical replicates, with error bars representing SEM. See also Figures S4 and S5.

exhibiting high levels of *in silico* caspase-3/7 activity within 24 h of treatment (Figures S4B–S4D). Additionally, the caspase activity of triple cultures in which the hiPSC-cFBs were substituted for hiPSC-dFBs were compared (Figure S4E). DNN caspase-3/7 analysis indicated no significant differences in caspase levels between the triple cultures.

As previously seen with the NKX2.5-$eGFP^{+}$ hESC-CMs (Figure 3G), caspase-3/7 activity was detected in the triple culture following the first treatment but only became apparent in the hiPSC-CM monoculture after the third round of Doxo exposure (Figure 4B). Further, *in silico* prediction of NKX2.5 confirmed that apoptosis in

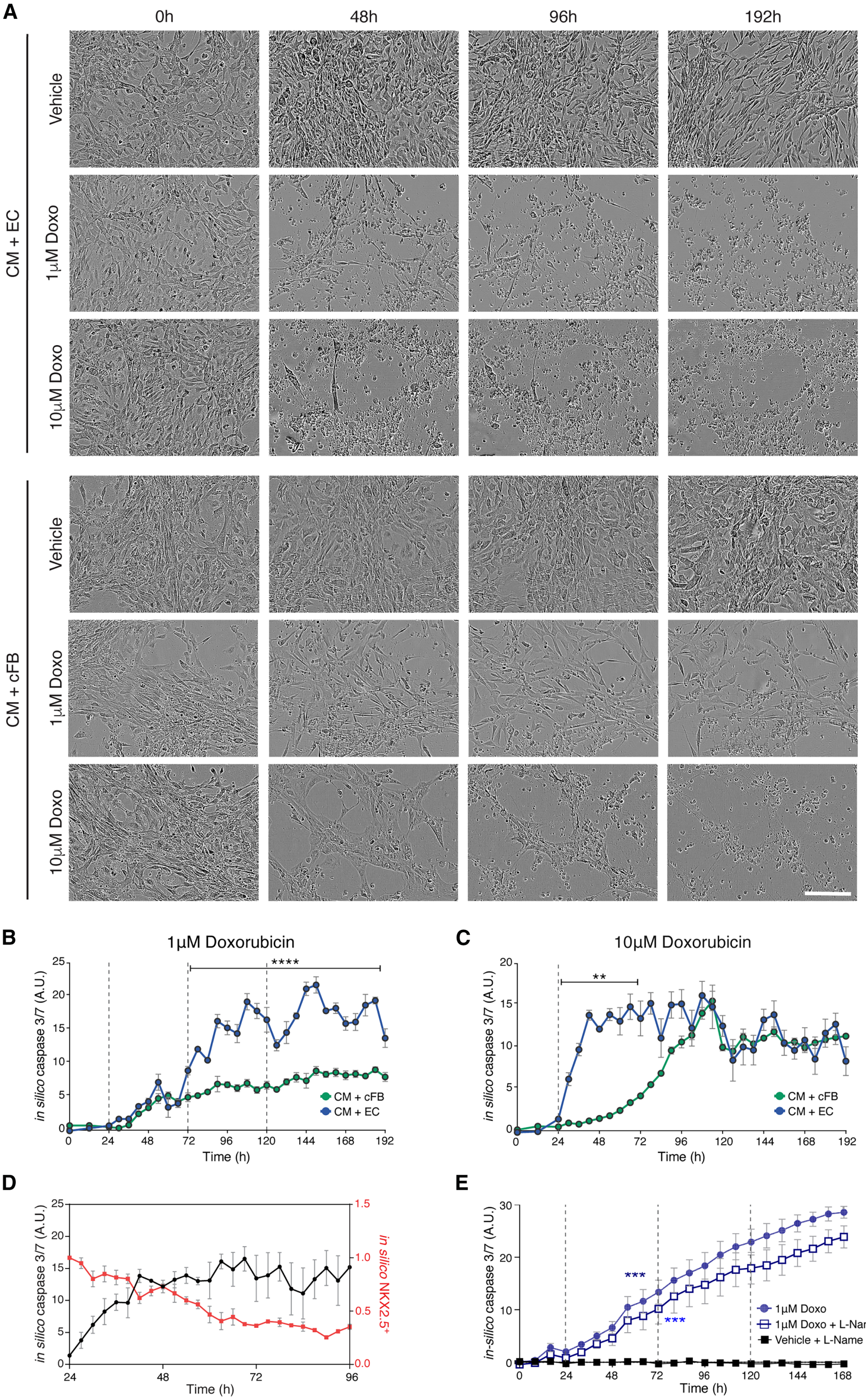

(legend on next page)

hiPSC-CMs occurred significantly faster when these cells were co-cultured with other cell types (Figure 4C). Detailed Doxo dose-response and time course analysis for *in silico* caspase-3/7 activity (Figure S5A) confirmed that repeated exposures to Doxo at concentrations below 1 μM resulted in minimal effects after 3 cycles across most conditions tested. An exception was monocultures of hiPSC-ECs, with noticeable caspase activity detected after 3 rounds of 0.03 μM Doxo treatment. At the higher concentration of 3 μM, Doxo induced significant caspase-3/7 activity from just 1 treatment cycle in hiPSC-CM and hiPSC-EC monocultures as well as in the multi-cell type culture, while hiPSC-cFBs required 2 cycles. Treatment with 10 μM Doxo quickly resulted in significant caspase-3/7 activity in hiPSC-EC and multi-cell-type cultures within 6 h of treatment, and within 2 days of the single treatment in monocultures of hiPSC-CMs and hiPSC-cFBs. In contrast, hiPSC-dFBs did not show consistent and significant caspase-3/7 activity at any Doxo concentration.

hiPSC-ECs amplify doxorubicin-induced cardiotoxicity in co-culture systems

Given the greater sensitivity of the hiPSC-ECs to Doxo, we hypothesized that these cells contributed to the increased caspase-3/7 activity observed in the multi-cell-type cultures. To test this, we treated dual-cell cultures of hiPSC-CMs with either ECs or cFBs to either a single exposure of 10 μM Doxo or the cumulative 1 μM treatment protocol. Phase contrast images showed that within 48 h of the initial Doxo treatment, many cells in the hiPSC-CM and hiPSC-EC co-cultures appeared apoptotic (Figure 5A). While cell death was also observed in the co-culture of hiPSC-CMs and hiPSC-cFBs, it was less pronounced and only evident under the 10 μM Doxo condition. This was quantitatively supported by the DNN caspase-3/7 analysis, which showed a significantly earlier caspase response in cultures containing hiPSC-ECs following 10 μM Doxo treatment and an overall significantly higher caspase activity signal in those exposed to the cumulative 1 μM Doxo treatment (Figures 5B and 5C).

Using the DNN tools, we also examined the specific effect on hiPSC-CMs within the co-cultures treated with 1 μM Doxo (Figure 5D). As previously observed, there was a sharp increase in caspase-3/7 activity (and general cell death) within the first 24 h of treatment that then plateaued. In contrast, the number of hiPSC-CMs steadily declined over the entire treatment period, suggesting that Doxo has an acute impact on hiPSC-ECs but more gradually affects the hiPSC-CMs. Overall, these results indicated interplay between the hiPSC-CMs and hiPSC-ECs in mediating Doxo-induced cardiotoxicity and suggest that ECs may play a pivotal role in exacerbating cardiomyocyte sensitivity to Doxo.

To investigate whether this EC-CM interplay involves paracrine signaling via nitric oxide (NO), we treated co-cultures of hiPSC-ECs and -CMs with Doxo in the presence or absence of the NO synthase (NOS) inhibitor L-NAME. DNN-based toxicity analysis showed that addition of 100 μM L-NAME to the 1 μM Doxo treatment delayed the onset of caspase-3/7 activation in hiPSC-CM-EC co-cultures by 24 h compared with Doxo alone (Figure 5E). A similar delay (44 h) was observed in hiPSC-EC monocultures, but not in hiPSC-CM monocultures (Figure S5B). This temporal shift in toxicity was also observed in co-cultures of ECs and CMs derived from independent hiPSC lines, where significant caspase-3/7 activation occurred 8 h later in cultures treated with L-NAME (Figure S5C).

DISCUSSION

Our experiments demonstrate that hiPSC-ECs accelerate the cardiotoxic effects of Doxo on co-cultured hPSC-CMs, underscoring the critical role of cell-cell interactions in drug-induced cardiotoxicity. Using ML-based tools that quantify caspase-3/7 activity from phase contrast images and can identify hPSC-CMs within mixed cultures, we achieved continuous, dye-free quantification of apoptosis

Figure 5. Contribution of hiPSC-ECs and -cFBs to Doxo-induced cardiotoxicity

(A) Representative phase contrast images of hiPSC-CMs co-cultured with isogenic hiPSC-ECs or hiPSC-cFBs treated with 1 μM or 10 μM Doxo. Images were acquired at time points corresponding to baseline (0 h), 24 h after treatments 1 and 2 (48 and 96 h, respectively), and the final time point (192 h). 10 μM Doxo was only administered once. Scale bar, 200 μm.

(B and C) *In silico* quantification of caspase-3/7 activity in either co-cultures of hiPSC-CMs with hiPSC-ECs or -cFBs and treated with either 1 μM (B) or 10 μM (C) Doxo. Dotted lines indicate the treatment time points, while the asterisks and black bars indicate the time points where there are statistically significant differences between the 2 co-cultures; $**p < 0.01$, $****p < 0.0001$.

(D) Simultaneous *in silico* quantification of caspase-3/7 activity (black line) and $NKX2.5^+$ cells (red line) in co-cultures of hiPSC-CMs and -ECs treated with 1 μM Doxo at 24 and 72 h time points, comparing the dynamics of apoptosis with rate of cardiomyocyte death.

(E) *In silico* quantification of caspase-3/7 activity in co-cultures of hiPSC-CMs with hiPSC-ECs treated with 1 μM Doxo with or without 100 μM L-NAME. Dotted lines indicate the treatment time points. Color-coded asterisks indicate the initial time point at which caspase-3/7 activity is significantly higher than baseline (0 h) for each condition. Statistical analysis was performed with two-way ANOVA. Analysis is based on 3 biological replicates, each with 2 or 3 technical replicates, with error bars representing SEM; $***p < 0.001$. See also Figure S5.

together with cell type-resolved readouts. The 2D format of our assay provided straightforward accessibility for imaging and analysis, allowing cumulative Doxo effects to be attributed to specific cell types and their interactions. Although this format does not replicate the full structural and physiological complexity of the human heart, it revealed paracrine-mediated crosstalk via NO signaling as a contributor to anthracycline toxicity.

Despite its effectiveness against a broad range of cancers, Doxo is well known for cardiotoxic side effects that can manifest either early or decades after treatment (Schimmel et al., 2004). The cumulative dose is a predictor of cardiac dysfunction, yet limiting the total dose primarily reduces instances of acute toxicity without diminishing the incidence of late-onset effects (Cappetta et al., 2018). The challenge of mitigating these effects, while maintaining therapeutic efficacy, underscores a pressing need for more accurate models to understand Doxo-induced cardiotoxicity and that of its analogs (van Gelder et al., 2023).

Advanced 3D cardiac models, including microtissues and EHTs, capture multicellular interactions and reveal rapid Doxo-induced functional impairment (Qiao et al., 2020; Schwach et al., 2024). However, they are less suited to real-time, cell type-specific readouts. Our 2D multi-cell-type culture complements these systems by isolating the contributions of key cardiac cell types to Doxo responses and enabling longitudinal, label-free quantification. Accordingly, we modeled cumulative exposure with repeated 1 μM, 4-h pulses every 48 h over 8 days. This scheme was based on the reported *in vivo* pharmacokinetics where the free-plasma concentration of Doxo rapidly drops from initial levels ranging between 20 nM and 2 μM and has a terminal half-life of 20–48 h (Barpe et al., 2010; Greene et al., 1983; Pang et al., 2013). Under these conditions, hPSC-CM monocultures required 3 treatment rounds for visible cell death, whereas co-cultures of hPSC-CMs, -ECs, and -cFBs showed signs of caspase-3/7 activation within 24 h of the first exposure, accompanied by earlier deterioration of contractile parameters. These findings demonstrate that intercellular interactions can modulate toxicity dynamics.

Our data are consistent with prior reports that cFBs and ECs exhibit early Doxo-induced stress responses. These studies identified a pro-fibrotic influence of Doxo on fibroblasts in rat hearts via TGF-β and SMAD3 signaling, triggering their transition to myofibroblasts (Cappetta et al., 2016). Furthermore, mouse cFBs exposed to Doxo were reported to become senescent leading to the secretion of pro-inflammatory cytokines, such as IL-1β (Espitia-Corredor et al., 2022). Likewise, clinical studies have associated Doxo cardiotoxicity with damage to the vascular endothelium (Bielak-Zmijewska et al., 2014; Wojcik et al., 2015), and a recent single-cell transcriptomic study reported a significant loss of ECs in mouse hearts following repeated intraperitoneal injections of Doxo (Huyan et al., 2024). Furthermore, both *in vitro* and animal studies have indicated that Doxo can compromise endothelial elasticity, increase cardiac microvasculature permeability, inhibit vascular network formation, and induce oxidative stress through altered reactive oxygen species and NO levels (Cappetta et al., 2018).

Consistent with ECs playing a role in Doxo-induced cardiotoxicity, pharmacologic NOS inhibition with L-NAME delayed the onset of caspase activity in hPSC-EC monocultures and in EC-CM co-cultures, but not in CM monocultures. This suggests that EC-derived NO (and/or downstream reactive nitrogen species) contributes to the priming of cardiomyocyte susceptibility to Doxo. Although L-NAME did not abolish toxicity, the shift in apoptosis timing supports NO as a contributing driver. These observations are in line with our previous work showing that EC-CM crosstalk via the NO pathway enhanced inflammatory responses and influenced contractility in hiPSC-based systems (Arslan et al., 2023). Together, these findings highlight EC-CM communication, and specifically NO-dependent signaling, as a modulator of anthracycline cardiotoxicity and demonstrate an experimentally accessible platform for further mechanistic dissection and intervention testing. Senescence and p53 signaling are also implicated in anthracycline cardiotoxicity (Linders et al., 2024). Although not assayed here, future investigations that incorporate senescence markers and targeted p53 perturbations into the label-free pipeline will clarify how these programs intersect with the NO-dependent EC-CM effects.

Live-cell imaging commonly relies on organic fluorophores or fluorescent proteins to monitor specific cellular processes or markers (Specht et al., 2017). However, dye signal decay, which can be exacerbated by culture medium changes, limits lengthy treatment protocols involving repeated dosing, as we observed with the caspase-3/7 reagent (Figure 2F). Tagging endogenous markers with fluorescent proteins can mitigate some of these issues but restricts assays to genetically engineered cell lines, thereby limiting the ability to assess patient-specific responses. To overcome these constraints, we applied a DNN trained for apoptosis analysis to quantify *in silico* caspase-3/7 activity from phase contrast images, enabling continuous label-free monitoring over extended periods. Moreover, the tool also segmented and classified hPSC-CMs within mixed cultures, facilitating cell type-resolved toxicodynamic analyses across multiple hPSC lines and co-culture contexts.

ML applications in cardiotoxicity assessment, particularly with hPSC-CMs, are advancing rapidly. Models trained on functional parameters, such as action potential, calcium cycling, or contraction properties, can distinguish

cardiotoxic from non-cardiotoxic compounds (Maddah et al., 2020; Pang et al., 2022; Sala et al., 2018; Serrano et al., 2023), and high-content imaging pipelines can quantify structural toxicity in fixed hiPSC-CMs (Grafton et al., 2021; Maddah et al., 2020). Notably, toxicity from continuous low dose (0.3–0.6 mM) Doxo exposure could be detected in phase contrast images of live hiPSC-CMs with greater sensitivity than cell confluency assays (Maddah et al., 2020). Our approach extends these advances by applying ML to repeated pulse exposure protocols with medium exchanges and by resolving apoptosis dynamics within multi-cell type cultures without fluorescent labels.

In summary, while hPSC-CMs have traditionally been the focus of *in vitro* cardiotoxicity studies, our data demonstrate that including other cardiac cell types, in particular hiPSC-ECs, critically influences Doxo cytotoxicity responses. Coupling a multi-cell-type human co-culture system with label-free ML analysis revealed EC-derived NO signaling as a contributor to the enhanced cardiotoxicity that was observed in hPSC-CM and -EC co-cultures. These findings highlight endothelial pathways as potential targets for mitigating anthracycline cardiotoxicity and exemplify how scalable human stem cell models can advance mechanism-driven drug safety testing.

METHODS

hPSC culture and differentiation

All hPSC lines were maintained as previously described (Brandao et al., 2020) in either Essential 8 medium (Thermo Fisher Scientific) or TeSR-E8 medium (STEMCELL Technologies) on vitronectin-coated plates. The following hPSC lines were used in this study: LUMC0020iCTRL-06 hiPSC line (RRID:CVCL_ZA25) (Zhang et al., 2014), alpha-actinin-2^{mEGFP} hiPSC line (RRID:CVCL_WM14), NCRM-1 hiPSC line genetically modified to constitutively express mCherry (Arslan et al., 2023), and HES-3 Mesp1^{mcherry}-NKX2.5^{eGFP} hESC line (RRID:CVCL_A8JT) (Den Hartogh et al., 2015).

The hPSC lines were differentiated into CMs, ECs, cFBs and dFBs following previously established protocols (Campostrini et al., 2021; Orlova et al., 2014; Itoh et al., 2013). All experiments were performed with cryopreserved, differentiated hPSCs that were thawed as previously described (van den Brink et al., 2020; Campostrini et al., 2021). Additional details regarding thawing and plating of the cells are provided in the supplemental methods.

Flow cytometry and immunofluorescence

Details regarding the preparation of the cells and staining procedure are provided in the supplemental methods. Table S1 lists antibodies used in this study.

Compound treatments

All compounds were reconstituted in DMSO according to the manufacturer's guidelines. Table S2 provides details of the drugs, including stock and final testing concentrations. Stock solutions were stored at -20°C, thawed only once on the day of use, and diluted in mBEL CM Maintenance Medium. For single treatments, hiPSC-CMs were exposed to the compounds for 30 min at the concentrations indicated in Table S2.

For the cumulative treatment protocol, Doxo was diluted in the culture medium appropriate for each cell type. Further details are provided in the supplemental methods. For NOS inhibition experiments, L-NAME remained present throughout the entire cumulative treatment period.

Live-cell imaging and dye-based caspase-3/7 detection

High-definition phase contrast images were captured at 3-h intervals using the Standard Adherent cell-by-cell scan type on an Incucyte S3 Live-Cell Analysis system (Sartorius). Per well, 5 areas were imaged, corresponding to ~80% of the total well area. Where applicable, NKX2.5-eGFP^{+} hESC-CMs images were acquired using the green fluorescence channel (300 ms acquisition).

For dye-based apoptosis detection, cells were incubated with Incucyte Caspase-3/7 reagents (Sartorius) prior to compound exposure, and fluorescence imaging was performed in parallel with phase contrast imaging at 3-h intervals. Further details regarding analysis are provided in the supplemental methods.

Contractility analysis

Bright-field videos were acquired on an Incucyte S3 system with modified custom acquisition software (Sartorius), immediately prior to compound addition and at 24-h intervals thereafter. Recordings (10–20 s at 100 fps) were made using a 10× objective. For analysis, each field of view was divided into 5 regions and saved as separate videos to reduce noise prior to processing. Contractile parameters were obtained using CardioMotion software (Stebbeds et al., 2023).

Analysis using *in silico* models

A DNN model was generated to identify hPSC-CMs. Phase contrast images were paired with corresponding NKX2.5 fluorescence images as ground truth. Image segmentation was performed using a U-Net-based architecture (Falk et al., 2019). A similar methodology was applied to train a distinct model for caspase-3/7 detection. Further details are provided in the supplemental methods.

Archived phase contrast image datasets and associated plate maps were exported from the Incucyte system and analyzed using the caspase-3/7 and NKX2.5 DNN models

(Sartorius). All images acquired during the experiments were included. Processing steps are described in the supplemental methods. *In silico* caspase-3/7 outputs were corrected to the vehicle control (DMSO) and normalized to 0 h. *In silico* NKX2.5$^+$ values were normalized to the initial time point (0 h).

Each experiment consisted of 3 biological replicates performed independently. Within each biological replicate, 2 or 3 technical replicate wells were included per condition, as indicated in the figure legends. Analyses were performed independently per biological replicate before pooling data for visualization and statistical testing.

Statistics

Statistical analyses were performed using Prism 9 (GraphPad). Data are presented as mean ± SEM. Time-course datasets were analyzed using a two-way repeated-measures ANOVA, with culture condition as the between-subject factor and time as the within-subject (repeated) factor. The Geisser-Greenhouse correction was applied where the assumption of sphericity was violated. Unless stated otherwise, Sidak's post-hoc test was used to adjust for multiple comparisons between conditions and across time points. Statistical significance was defined as $p < 0.05$ and represented as *$p < 0.05$, **$p < 0.01$, ***$p < 0.001$, or ****$p < 0.0001$.

RESOURCE AVAILABILITY

Lead contact

Further information and resource requests should be directed to Dr. Richard Davis (r.p.davis@lumc.nl).

Materials availability

This study did not generate any new reagents.

Data and code availability

- The raw datasets have been deposited in Zenodo (https://doi.org/10.5281/zenodo.17974612). All other requests should be directed to the lead contact (r.p.davis@lumc.nl).

ACKNOWLEDGMENTS

We thank F.E. van den Hill for providing endothelial cells. Research support and funding for this study were provided by Sartorius AG, as acknowledged by authors C.L.M., B.J.v.M., R.P.D., and M.B. Additional funding support came from a research grant (CARMEN; LSHM20018) co-funded by the PPP Allowance made available by Health~Holland TKI-LSH to stimulate public-private partnerships, a Novo Nordisk Foundation grant (NNF21CC0073729; reNEW), and an NWO Gravitation project funded by the Ministry of Education, Culture, and Science of the government of the Netherlands (024.003.001). K.R. is Chargé de Recherche at the Institut National de la Santé et de la Recherche Médicale (INSERM). The graphical abstract was created in https://BioRender.com.

AUTHOR CONTRIBUTIONS

Conceptualization, M.B., C.L.M., B.J.v.M., R.P.D., R.W., and R.S.; methodology, M.B., B.J.v.M., R.P.D., R.S., and K.B.; software, E.S.P. and P.K.; formal analysis and investigation, M.B., J.G., R.S., E.S.P., and A.C.; resources, M.P.H.M., K.R., V.O., P.K., and K.B.; writing – original draft preparation, M.B. and R.P.D.; writing – review and editing, M.B., C.L.M., B.J.v.M., and R.P.D.; funding acquisition, C.L.M., B.J.v.M., R.P.D., and R.W.; supervision: C.L.M., B.J.v.M., R.P.D., R.W., J.A., and M.W.O.

DECLARATION OF INTERESTS

C.L.M. has advisory roles in HeartBeat.bio AG, Angios GmBH, Mogrify Limited, Cellistic, and Sartorius AG. E.S.P., P.K., A.C., R.S., K.B., R.W., J.A., and M.W.O. were all employees of Sartorius AG at the time the study was conducted. A patent related to this work (EP4145385) was filed.

SUPPLEMENTAL INFORMATION

Supplemental information can be found online at https://doi.org/10.1016/j.stemcr.2026.102816.

Received: December 19, 2025
Revised: January 14, 2026
Accepted: January 15, 2026
Published: February 12, 2026

REFERENCES

Arslan, U., Brescia, M., Meraviglia, V., Nahon, D.M., van Helden, R.W.J., Stein, J.M., van den Hil, F.E., van Meer, B.J., Vila Cuenca, M., Mummery, C.L., et al. (2023). Vascularized hiPSC-derived 3D cardiac microtissue on chip. Stem Cell Rep. *18*, 1394–1404. https://doi.org/10.1016/j.stemcr.2023.06.001.

Barpe, D.R., Rosa, D.D., and Froehlich, P.E. (2010). Pharmacokinetic evaluation of doxorubicin plasma levels in normal and overweight patients with breast cancer and simulation of dose adjustment by different indexes of body mass. Eur. J. Pharm. Sci. *41*, 458–463. https://doi.org/10.1016/j.ejps.2010.07.015.

Bielak-Zmijewska, A., Wnuk, M., Przybylska, D., Grabowska, W., Lewinska, A., Alster, O., Korwek, Z., Cmoch, A., Myszka, A., Pikula, S., et al. (2014). A comparison of replicative senescence and doxorubicin-induced premature senescence of vascular smooth muscle cells isolated from human aorta. Biogerontology *15*, 47–64. https://doi.org/10.1007/s10522-013-9477-9.

Brandao, K.O., van den Brink, L., Miller, D.C., Grandela, C., van Meer, B.J., Mol, M.P.H., de Korte, T., Tertoolen, L.G.J., Mummery, C.L., Sala, L., et al. (2020). Isogenic Sets of hiPSC-CMs Harboring Distinct KCNH2 Mutations Differ Functionally and in Susceptibility to Drug-Induced Arrhythmias. Stem Cell Rep. *15*, 1127–1139. https://doi.org/10.1016/j.stemcr.2020.10.005.

Campostrini, G., Meraviglia, V., Giacomelli, E., van Helden, R.W.J., Yiangou, L., Davis, R.P., Bellin, M., Orlova, V.V., and Mummery, C.L. (2021). Generation, functional analysis and applications of

isogenic three-dimensional self-aggregating cardiac microtissues from human pluripotent stem cells. Nat. Protoc. *16*, 2213–2256. https://doi.org/10.1038/s41596-021-00497-2.

Cappetta, D., Esposito, G., Piegari, E., Russo, R., Ciuffreda, L.P., Rivellino, A., Berrino, L., Rossi, F., De Angelis, A., and Urbanek, K. (2016). SIRT1 activation attenuates diastolic dysfunction by reducing cardiac fibrosis in a model of anthracycline cardiomyopathy. Int. J. Cardiol. *205*, 99–110. https://doi.org/10.1016/j.ijcard.2015.12.008.

Cappetta, D., Rossi, F., Piegari, E., Quaini, F., Berrino, L., Urbanek, K., and De Angelis, A. (2018). Doxorubicin targets multiple players: A new view of an old problem. Pharmacol. Res. *127*, 4–14. https://doi.org/10.1016/j.phrs.2017.03.016.

Csapo, M., and Lazar, L. (2014). Chemotherapy-Induced Cardiotoxicity: Pathophysiology and Prevention. Clujul Med. *87*, 135–142. https://doi.org/10.15386/cjmed-339.

de Korte, T., Katili, P.A., Mohd Yusof, N.A.N., van Meer, B.J., Saleem, U., Burton, F.L., Smith, G.L., Clements, P., Mummery, C.L., Eschenhagen, T., et al. (2020). Unlocking Personalized Biomedicine and Drug Discovery with Human Induced Pluripotent Stem Cell-Derived Cardiomyocytes: Fit for Purpose or Forever Elusive? Annu. Rev. Pharmacol. Toxicol. *60*, 529–551. https://doi.org/10.1146/annurev-pharmtox-010919-023309.

Den Hartogh, S.C., Schreurs, C., Monshouwer-Kloots, J.J., Davis, R.P., Elliott, D.A., Mummery, C.L., and Passier, R. (2015). Dual reporter MESP1 mCherry/w-NKX2-5 eGFP/w hESCs enable studying early human cardiac differentiation. Stem Cell. *33*, 56–67. https://doi.org/10.1002/stem.1842.

Elliott, D.A., Braam, S.R., Koutsis, K., Ng, E.S., Jenny, R., Lagerqvist, E.L., Biben, C., Hatzistavrou, T., Hirst, C.E., Yu, Q.C., et al. (2011). NKX2-5 eGFP/w hESCs for isolation of human cardiac progenitors and cardiomyocytes. Nat. Methods *8*, 1037–1040. https://doi.org/10.1038/nmeth.1740.

Espitia-Corredor, J.A., Shamoon, L., Olivares-Silva, F., Rimassa-Taré, C., Muñoz-Rodríguez, C., Espinoza-Pérez, C., Sánchez-Ferrer, C.F., Peiró, C., and Díaz-Araya, G. (2022). Resolvin E1 attenuates doxorubicin-induced cardiac fibroblast senescence: A key role for IL-1beta. Biochim. Biophys. Acta. Mol. Basis Dis. *1868*, 166525. https://doi.org/10.1016/j.bbadis.2022.166525.

Falk, T., Mai, D., Bensch, R., Çiçek, Ö., Abdulkadir, A., Marrakchi, Y., Böhm, A., Deubner, J., Jäckel, Z., Seiwald, K., et al. (2019). U-Net: deep learning for cell counting, detection, and morphometry. Nat. Methods *16*, 67–70. https://doi.org/10.1038/s41592-018-0261-2.

Georgiopoulos, G., Makris, N., Laina, A., Theodorakakou, F., Briasoulis, A., Trougakos, I.P., Dimopoulos, M.A., Kastritis, E., and Stamatelopoulos, K. (2023). Cardiovascular Toxicity of Proteasome Inhibitors: Underlying Mechanisms and Management Strategies. JACC. CardioOncol. *5*, 1–21. https://doi.org/10.1016/j.jaccao.2022.12.005.

Giacomelli, E., Meraviglia, V., Campostrini, G., Cochrane, A., Cao, X., van Helden, R.W.J., Krotenberg Garcia, A., Mircea, M., Kostidis, S., Davis, R.P., et al. (2020). Human-iPSC-Derived Cardiac Stromal Cells Enhance Maturation in 3D Cardiac Microtissues and Reveal Non-cardiomyocyte Contributions to Heart Disease. Cell Stem Cell *26*, 862–879.e11. https://doi.org/10.1016/j.stem.2020.05.004.

Grafton, F., Ho, J., Ranjbarvaziri, S., Farshidfar, F., Budan, A., Steltzer, S., Maddah, M., Loewke, K.E., Green, K., Patel, S., et al. (2021). Deep learning detects cardiotoxicity in a high-content screen with induced pluripotent stem cell-derived cardiomyocytes. eLife *10*, e68714. https://doi.org/10.7554/eLife.68714.

Greene, R.F., Collins, J.M., Jenkins, J.F., Speyer, J.L., and Myers, C.E. (1983). Plasma pharmacokinetics of adriamycin and adriamycinol: implications for the design of in vitro experiments and treatment protocols. Cancer Res. *43*, 3417–3421.

Huyan, Y., Chen, X., Chang, Y., Hua, X., Fan, X., Shan, D., Xu, Z., Tao, M., Zhang, H., Liu, S., and Song, J. (2024). Single-cell transcriptomic analysis reveals myocardial fibrosis mechanism of doxorubicin-induced cardiotoxicity. Int. Heart J. *65*, 487–497. https://doi.org/10.1536/ihj.23-302.

Itoh, M., Umegaki-Arao, N., Guo, Z., Liu, L., Higgins, C.A., and Christiano, A.M. (2013). Generation of 3D skin equivalents fully reconstituted from human induced pluripotent stem cells (iPSCs). PLoS One *8*, e77673. https://doi.org/10.1371/journal.pone.0077673.

Karabulut, D., Ozturk, E., Kaymak, E., Akin, A.T., and Yakan, B. (2021). Thymoquinone attenuates doxorubicin-cardiotoxicity in rats. J. Biochem. Mol. Toxicol. *35*, e22618. https://doi.org/10.1002/jbt.22618.

Kettenhofen, R., and Bohlen, H. (2008). Preclinical assessment of cardiac toxicity. Drug Discov. Today *13*, 702–707. https://doi.org/10.1016/j.drudis.2008.06.011.

Linders, A.N., Dias, I.B., Lopez Fernandez, T., Tocchetti, C.G., Bomer, N., and Van der Meer, P. (2024). A review of the pathophysiological mechanisms of doxorubicin-induced cardiotoxicity and aging. NPJ Aging *10*, 9. https://doi.org/10.1038/s41514-024-00135-7.

Maddah, M., Mandegar, M.A., Dame, K., Grafton, F., Loewke, K., and Ribeiro, A.J.S. (2020). Quantifying drug-induced structural toxicity in hepatocytes and cardiomyocytes derived from hiPSCs using a deep learning method. J. Pharmacol. Toxicol. Methods *105*, 106895. https://doi.org/10.1016/j.vascn.2020.106895.

Mak, I.W., Evaniew, N., and Ghert, M. (2014). Lost in translation: animal models and clinical trials in cancer treatment. Am. J. Transl. Res. *6*, 114–118.

Michihiko, U., Yoshihiko, K., Koh-ichi, Y., Nobuyuki, M., Motoyuki, I., Takashi, M., and Iwao, Y. (2006). Doxorubicin induces apoptosis by activation of caspase-3 in cultured cardiomyocytes in vitro and rat cardiac ventricles in vivo. J. Pharmacol. Sci. *101*, 151–158. https://doi.org/10.1254/jphs.FP0050980.

Orlova, V.V., van den Hil, F.E., Petrus-Reurer, S., Drabsch, Y., Ten Dijke, P., and Mummery, C.L. (2014). Generation, expansion and functional analysis of endothelial cells and pericytes derived from human pluripotent stem cells. Nat. Protoc. *9*, 1514–1531. https://doi.org/10.1038/nprot.2014.102.

Pang, B., Qiao, X., Janssen, L., Velds, A., Groothuis, T., Kerkhoven, R., Nieuwland, M., Ovaa, H., Rottenberg, S., van Tellingen, O., et al. (2013). Drug-induced histone eviction from open chromatin

contributes to the chemotherapeutic effects of doxorubicin. Nat. Commun. *4*, 1908. https://doi.org/10.1038/ncomms2921.

Pang, J.K.S., Chia, S., Zhang, J., Szyniarowski, P., Stewart, C., Yang, H., Chan, W.K., Ng, S.Y., and Soh, B.S. (2022). Characterizing arrhythmia using machine learning analysis of Ca(2+) cycling in human cardiomyocytes. Stem Cell Rep. *17*, 1810–1823. https://doi.org/10.1016/j.stemcr.2022.06.005.

Qiao, X., van der Zanden, S.Y., Wander, D.P.A., Borràs, D.M., Song, J.Y., Li, X., van Duikeren, S., van Gils, N., Rutten, A., van Herwaarden, T., et al. (2020). Uncoupling DNA damage from chromatin damage to detoxify doxorubicin. Proc. Natl. Acad. Sci. USA *117*, 15182–15192. https://doi.org/10.1073/pnas.1922072117.

Raniga, K., Nasir, A., Vo, N.T.N., Vaidyanathan, R., Dickerson, S., Hilcove, S., Mosqueira, D., Mirams, G.R., Clements, P., Hicks, R., et al. (2024). Strengthening cardiac therapy pipelines using human pluripotent stem cell-derived cardiomyocytes. Cell Stem Cell *31*, 292–311. https://doi.org/10.1016/j.stem.2024.01.007.

Rawat, P.S., Jaiswal, A., Khurana, A., Bhatti, J.S., and Navik, U. (2021). Doxorubicin-induced cardiotoxicity: An update on the molecular mechanism and novel therapeutic strategies for effective management. Biomed. Pharmacother. *139*, 111708. https://doi.org/10.1016/j.biopha.2021.111708.

Sala, L., van Meer, B.J., Tertoolen, L.G.J., Bakkers, J., Bellin, M., Davis, R.P., Denning, C., Dieben, M.A.E., Eschenhagen, T., Giacomelli, E., et al. (2018). MUSCLEMOTION: A Versatile Open Software Tool to Quantify Cardiomyocyte and Cardiac Muscle Contraction In Vitro and In Vivo. Circ. Res. *122*, e5–e16. https://doi.org/10.1161/CIRCRESAHA.117.312067.

Saleem, U., van Meer, B.J., Katili, P.A., Mohd Yusof, N.A.N., Mannhardt, I., Garcia, A.K., Tertoolen, L., de Korte, T., Vlaming, M.L.H., McGlynn, K., et al. (2020). Blinded, Multicenter Evaluation of Drug-induced Changes in Contractility Using Human-induced Pluripotent Stem Cell-derived Cardiomyocytes. Toxicol. Sci. *176*, 103–123. https://doi.org/10.1093/toxsci/kfaa058.

Sapia, L., Palomeque, J., Mattiazzi, A., and Petroff, M.V. (2010). Na+/K+-ATPase inhibition by ouabain induces CaMKII-dependent apoptosis in adult rat cardiac myocytes. J. Mol. Cell. Cardiol. *49*, 459–468. https://doi.org/10.1016/j.yjmcc.2010.04.013.

Schimmel, K.J.M., Richel, D.J., van den Brink, R.B.A., and Guchelaar, H.J. (2004). Cardiotoxicity of cytotoxic drugs. Cancer Treat Rev. *30*, 181–191. https://doi.org/10.1016/j.ctrv.2003.07.003.

Schwach, V., Slaats, R.H., Cofiño-Fabres, C., Ten Den, S.A., Rivera-Arbeláez, J.M., Dannenberg, M., van Boheemen, C., Ribeiro, M.C., van der Zanden, S.Y., Nollet, E.E., et al. (2024). A safety screening platform for individualized cardiotoxicity assessment. iScience *27*, 109139. https://doi.org/10.1016/j.isci.2024.109139.

Serrano, R., Feyen, D.A.M., Bruyneel, A.A.N., Hnatiuk, A.P., Vu, M.M., Amatya, P.L., Perea-Gil, I., Prado, M., Seeger, T., Wu, J.C., et al. (2023). A deep learning platform to assess drug proarrhythmia risk. Cell Stem Cell *30*, 86–95.e4. https://doi.org/10.1016/j.stem.2022.12.002.

Specht, E.A., Braselmann, E., and Palmer, A.E. (2017). A Critical and Comparative Review of Fluorescent Tools for Live-Cell Imaging. Annu. Rev. Physiol. *79*, 93–117. https://doi.org/10.1146/annurev-physiol-022516-034055.

Stebbeds, W., Raniga, K., Standing, D., Wallace, I., Bayliss, J., Brown, A., Kasprowicz, R., Dalmas Wilk, D., Deakyne, J., Clements, P., et al. (2023). CardioMotion: identification of functional and structural cardiotoxic liabilities in small molecules through bright-field kinetic imaging. Toxicol. Sci. *195*, 61–70. https://doi.org/10.1093/toxsci/kfad065.

van den Brink, L., Brandão, K.O., Yiangou, L., Mol, M.P.H., Grandela, C., Mummery, C.L., Verkerk, A.O., and Davis, R.P. (2020). Cryopreservation of human pluripotent stem cell-derived cardiomyocytes is not detrimental to their molecular and functional properties. Stem Cell Res. *43*, 101698. https://doi.org/10.1016/j.scr.2019.101698.

van der Pal, H.J., van Dalen, E.C., van Delden, E., van Dijk, I.W., Kok, W.E., Geskus, R.B., Sieswerda, E., Oldenburger, F., Koning, C.C., van Leeuwen, F.E., et al. (2012). High risk of symptomatic cardiac events in childhood cancer survivors. J. Clin. Oncol. *30*, 1429–1437. https://doi.org/10.1200/JCO.2010.33.4730.

van Gelder, M.A., van der Zanden, S.Y., Vriends, M.B.L., Wagensveld, R.A., van der Marel, G.A., Codée, J.D.C., Overkleeft, H.S., Wander, D.P.A., and Neefjes, J.J.C. (2023). Re-Exploring the Anthracycline Chemical Space for Better Anti-Cancer Compounds. J. Med. Chem. *66*, 11390–11398. https://doi.org/10.1021/acs.jmedchem.3c00853.

Wojcik, T., Buczek, E., Majzner, K., Kolodziejczyk, A., Miszczyk, J., Kaczara, P., Kwiatek, W., Baranska, M., Szymonski, M., and Chlopicki, S. (2015). Comparative endothelial profiling of doxorubicin and daunorubicin in cultured endothelial cells. Toxicol. Vitro *29*, 512–521. https://doi.org/10.1016/j.tiv.2014.12.009.

Zaragoza, C., Gomez-Guerrero, C., Martin-Ventura, J.L., Blanco-Colio, L., Lavin, B., Mallavia, B., Tarin, C., Mas, S., Ortiz, A., and Egido, J. (2011). Animal models of cardiovascular diseases. J. Biomed. Biotechnol. *2011*, 497841. https://doi.org/10.1155/2011/497841.

Zhang, M., D'Aniello, C., Verkerk, A.O., Wrobel, E., Frank, S., Ward-van Oostwaard, D., Piccini, I., Freund, C., Rao, J., Seebohm, G., et al. (2014). Recessive cardiac phenotypes in induced pluripotent stem cell models of Jervell and Lange-Nielsen syndrome: disease mechanisms and pharmacological rescue. Proc. Natl. Acad. Sci. USA *111*, E5383–E5392. https://doi.org/10.1073/pnas.1419553111.

Zushin, P.J.H., Mukherjee, S., and Wu, J.C. (2023). FDA Modernization Act 2.0: transitioning beyond animal models with human cells, organoids, and AI/ML-based approaches. J. Clin. Investig. *133*, e175824. https://doi.org/10.1172/JCI175824.

Stem Cell Reports, Volume *21*

Supplemental Information

A simplified co-culture reveals altered cardiotoxic responses to doxorubicin in hPSC-derived cardiomyocytes in the presence of endothelial cells

Marcella Brescia, James Gallant, Andrea Chatrian, Paul Keselman, Elsa Sörman Paulsson, Mervyn P.H. Mol, Rickard Sjögren, Karine Raymond, Valeria Orlova, Kalpana Barnes, Richard Wales, Jonas Austerjost, Michael W. Olszowy, Christine L. Mummery, Berend J. van Meer, and Richard P. Davis

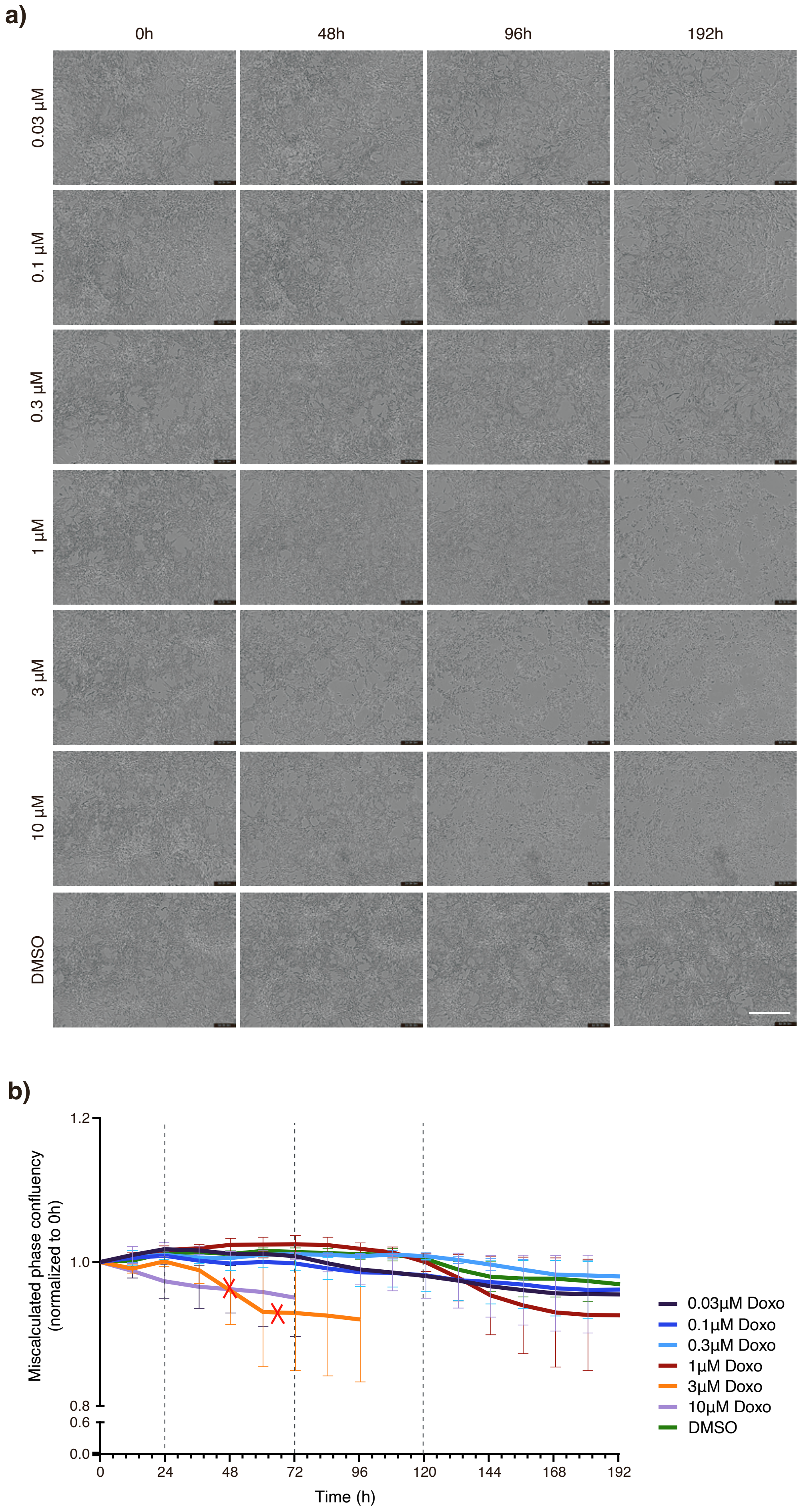

a)
0h
48h
96h
192h
0.03 µM
0.1 µM
0.3 µM
1 µM
3 µM
10 µM
DMSO
b)
Miscalculated phase confluency (normalized to 0h)
1.2
1.0
0.8
0.6
0.0
0
24
48
72
96
120
144
168
192
Time (h)
0.03µM Doxo
0.1µM Doxo
0.3µM Doxo
1µM Doxo
3µM Doxo
10µM Doxo
DMSO

Figure S1. Effect of cumulative Doxo treatment on hiPSC-CMs, related to Figure 1.

a) Representative phase contrast images of hiPSC-CM monocultures treated cumulatively with various Doxo concentrations or DMSO (vehicle control). Images were acquired at baseline (0h), 24 h after the first and second treatments (48h and 96h, respectively), and at the final time point (192h). All treatments followed the cumulative dosing protocol outlined in Fig. 1, except 10 µM Doxo, which was administered as a single exposure. Scale bar, 400µm.

b) Incucyte-based quantification of phase confluency (percentage of the image area covered by objects), normalized to baseline (0 h). Red crosses indicate time points at which all cells were visually assessed as non-viable. Dotted lines indicate Doxo treatment time points. Data represent mean ± SEM (*n* = 3 biological replicates, each with 3 technical replicates).

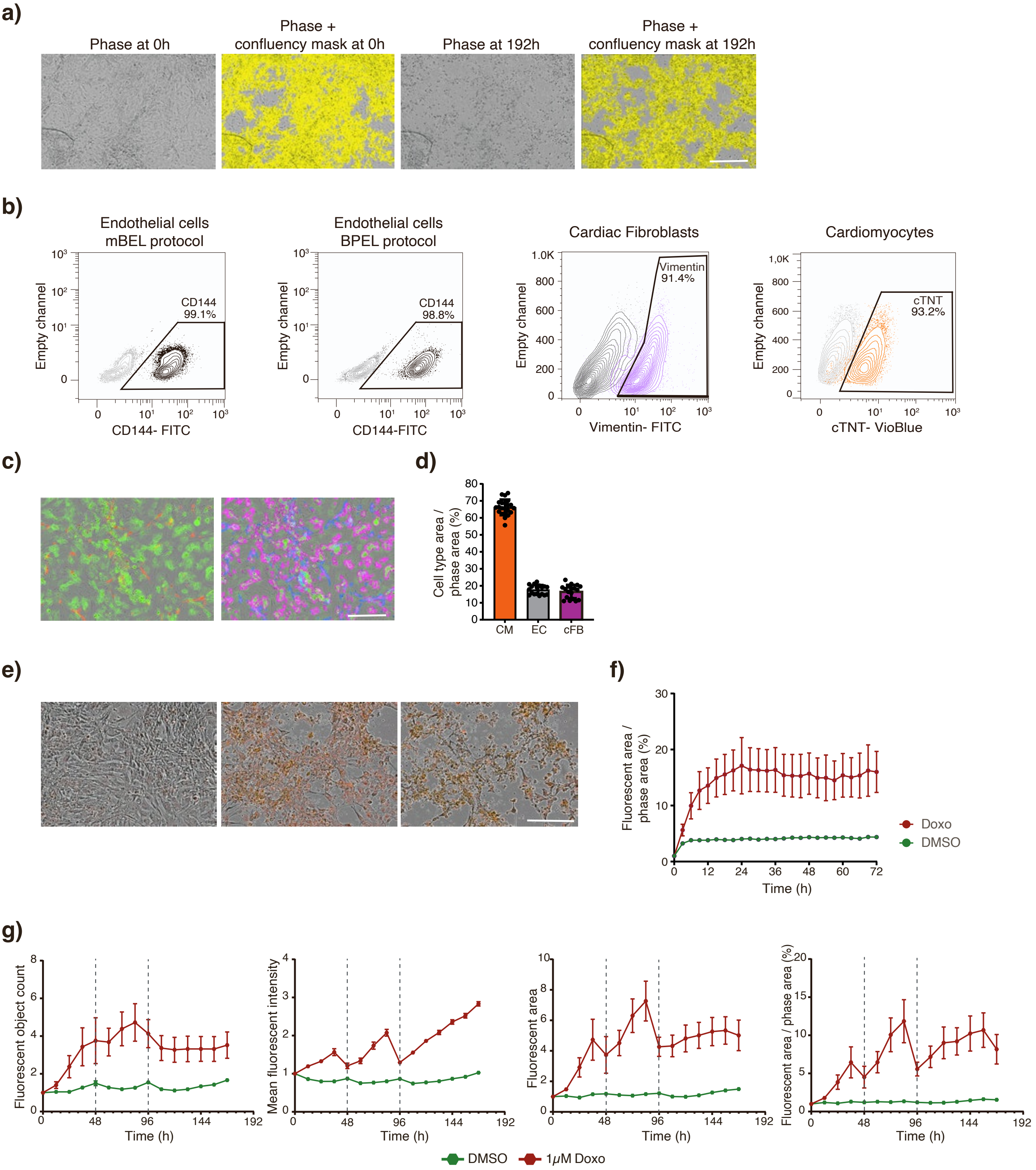
a)
Phase at 0h
Phase + confluency mask at 0h
Phase at 192h
Phase + confluency mask at 192h
b)
Endothelial cells mBEL protocol
Endothelial cells BPEL protocol
Cardiac Fibroblasts
Cardiomyocytes
Empty channel
CD144 99.1%
CD144 98.8%
Vimentin 91.4%
cTNT 93.2%
CD144- FITC
CD144-FITC
Vimentin- FITC
cTNT- VioBlue
c)
d)
Cell type area / phase area (%)
CM
EC
cFB
e)
f)
Fluorescent area / phase area (%)
Doxo
DMSO
Time (h)
g)
Fluorescent object count
Mean fluorescent intensity
Fluorescent area
Fluorescent area / phase area (%)
Time (h)
DMSO
1μM Doxo

Figure S2. Characterization of hiPSC-derived cardiac cell types and limitations of confluency- and fluorescence-based toxicity readouts, related to Figures 1 and 2.

a) Representative phase contrast images of multi-cell type cultures (hiPSC-CMs, -ECs, and -cFBs) with the corresponding phase confluency masks (highlighted in yellow). Masks illustrate the inability of confluency-based metrics to distinguish live from dead cells. Images are shown at baseline (0h) and at 192 h following cumulative 1 µM Doxo treatment, when all cells were visually non-viable. Scale bar, 400 µm.

b) Flow cytometric characterization of hiPSC-derived cell types. Representative plots showing hiPSC-ECs generated using either mBEL- or bPEL-based differentiation protocols expressing CD144, hiPSC-cFBs expressing vimentin and hiPSC-CMs expressing cardiac troponin T (cTnT).

c) Representative merged phase, green and red fluorescence image (*left*) showing the distribution of cell types in a multi-cell type culture (hiPSC-CMs, α-actinin mEGFP$^+$; hiPSC-ECs, mCherry$^+$; hiPSC-cFBs, non-fluorescent), and the corresponding segmentation masks (*right*) used to quantify each cell type. Scale bar, 400 µm.

d) Proportion of each cell type present in multi-cell type cultures at baseline (0h), based on segmentation of the masks shown in **(c)**. *N* = 18 wells analyzed.

e) Merged phase and red fluorescence images of hiPSC-CMs labelled with caspase 3/7 dye and treated once with 10 µM Doxo. Images correspond to baseline (0h), 24 h and 72 h after treatment. Scale bar, 200 µm.

f) Time-course quantification of caspase 3/7 fluorescence area relative to phase contrast area (confluency mask) in hiPSC-CMs treated with 10 µM Doxo or DMSO (vehicle control). Data represent mean ± SEM (*n* = 3 technical replicates).

g) Evaluation of different fluorescence-based metrics for quantifying temporal changes in caspase 3/7 in multi-cell type cultures undergoing cumulative 1 µM Doxo or DMSO treatment. Dotted lines indicate the treatment time points. All values were normalized to baseline (0 h). Data represent mean ± SEM (*n* = 3 technical replicates).

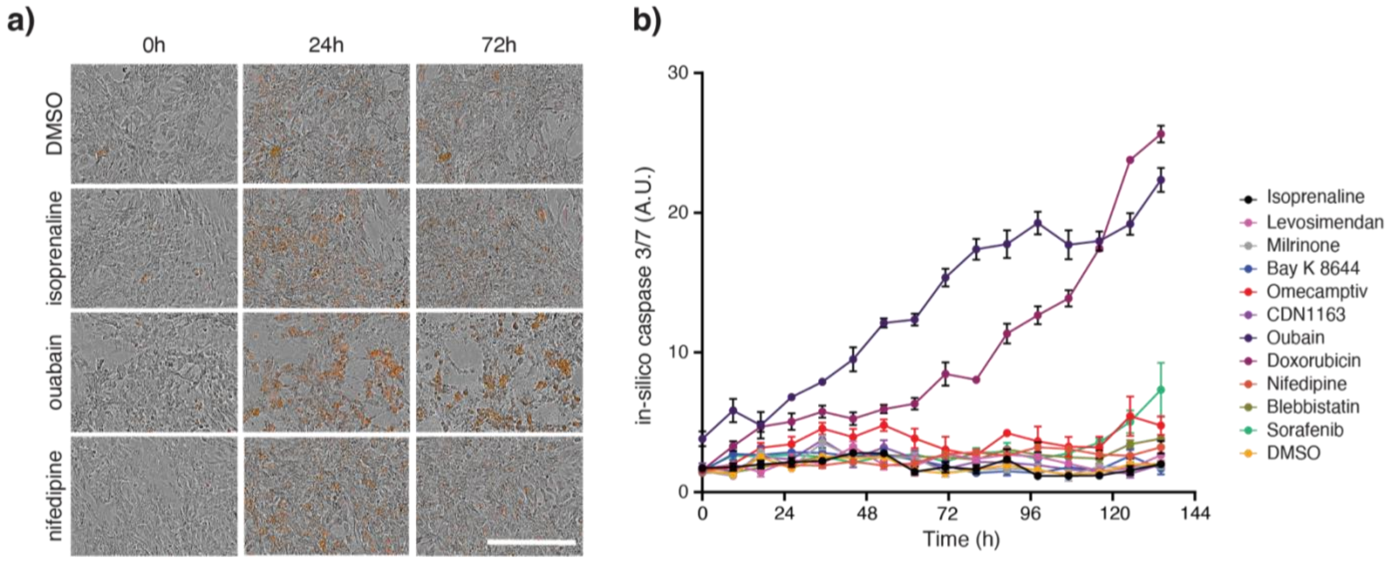

Figure S3. Analysis of caspase 3/7 activity in hiPSC-CMs following treatment with various compounds, related to Figure 3.

a) Merged phase and red fluorescence images of hiPSC-CMs labelled with caspase 3/7 dye and treated once with the indicated compounds. Time points correspond to baseline (0h), 24 h and 72 h after treatment. Scale bar, 400 µm.

b) *In silico* quantification of caspase 3/7 activity over time in hiPSC-CMs treated once with indicated compounds. The treatment concentrations for the compounds are listed in Supplementary Table 2. Data represent mean ± SEM (*n* = 3 biological replicates, each with 3 technical replicates).

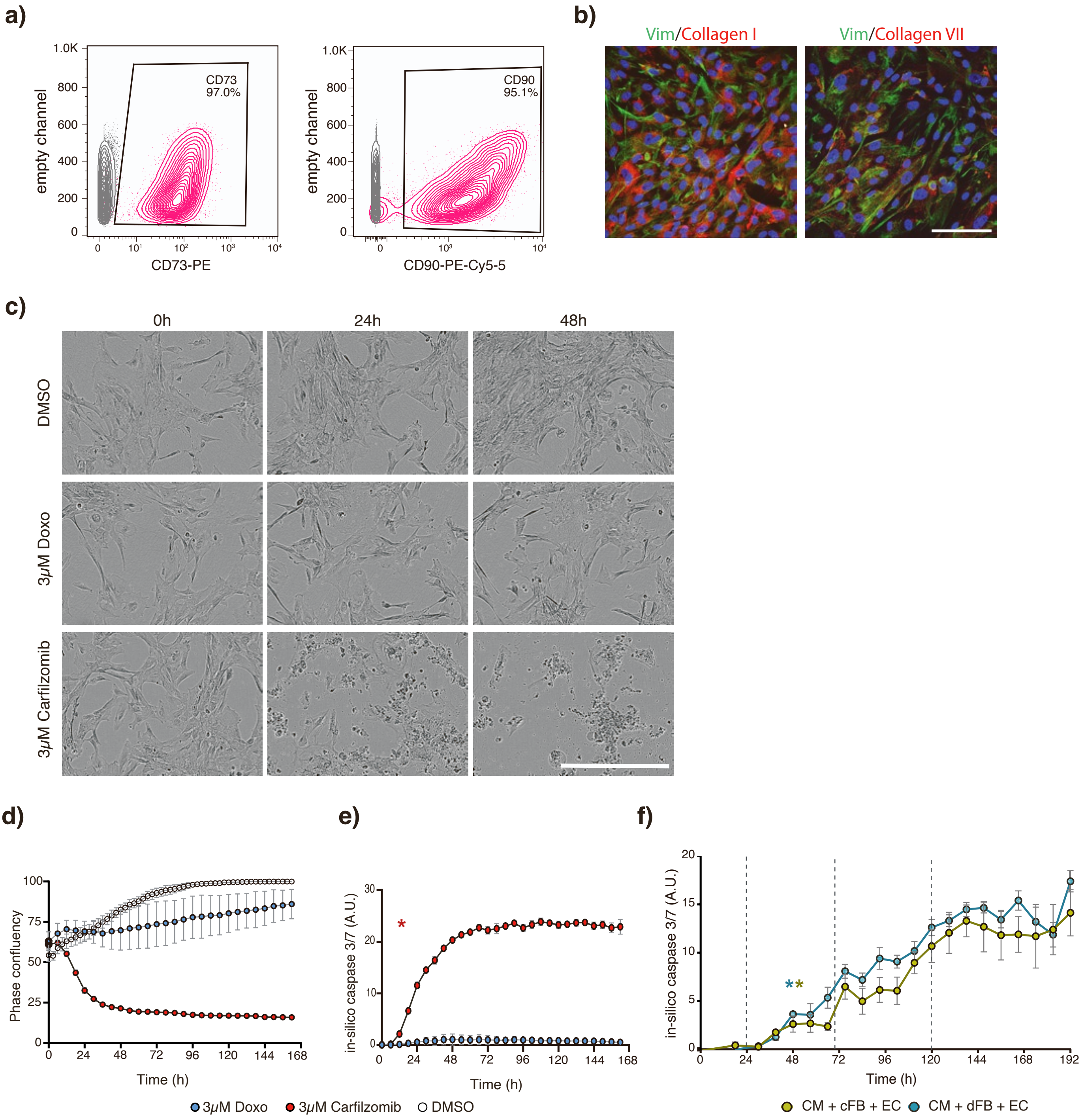

a)
b)
Vim/Collagen I
Vim/Collagen VII
empty channel
CD73
97.0%
CD73-PE
CD90
95.1%
CD90-PE-Cy5-5
c)
0h
24h
48h
DMSO
3μM Doxo
3μM Carfilzomib
d)
e)
f)
Phase confluency
in-silico caspase 3/7 (A.U.)
Time (h)
3μM Doxo
3μM Carfilzomib
DMSO
CM + cFB + EC
CM + dFB + EC

Figure S4. Characterization of hiPSC-dFBs and their response to cardiotoxic compounds, related to Figure 4.

a) Representative flow cytometry analysis of hiPSC-dFBs expressing the fibroblast surface markers CD73 and CD90.

b) Representative immunofluorescence images showing expression of the fibroblast marker vimentin and extracellular matrix proteins collagen I and collagen VII in hiPSC-dFB**.** Scale bar, 100 µm.

c) Representative phase contrast images of hiPSC-dFBs treated with vehicle control (3 µM DMSO), 3 µM Doxo or 3 µM Carfilzomib. Images were acquired at baseline (0h), and 24 h and 48 h after treatment. Scale bar, 400 µm.

d) Time course quantification of phase confluency area calculated from live cell imaging for the treatment conditions described in **(c)**.

e) *In silico* quantification of caspase 3/7 activity in hiPSC-dFbs for the treatment conditions described in **(c)**, normalized to the vehicle control. The asterisk indicates the first time point at which caspase 3/7 activity was significantly higher than baseline (0 h).

f) *In silico* quantification of caspase 3/7 activity in multi-cell type culture conditions containing either hiPSC-cFBs or hiPSC-dFBs undergoing cumulative 1 µM Doxo treatment (dotted lines). Asterisks indicate the first time point at which caspase 3/7 activity was significantly higher than baseline (0 h).

Statistical significance was determined using two-way repeated measures ANOVA with Sidak's multiple comparison test. Data represent mean ± SEM (*n* = 3 biological replicates, each with 3 technical replicates).

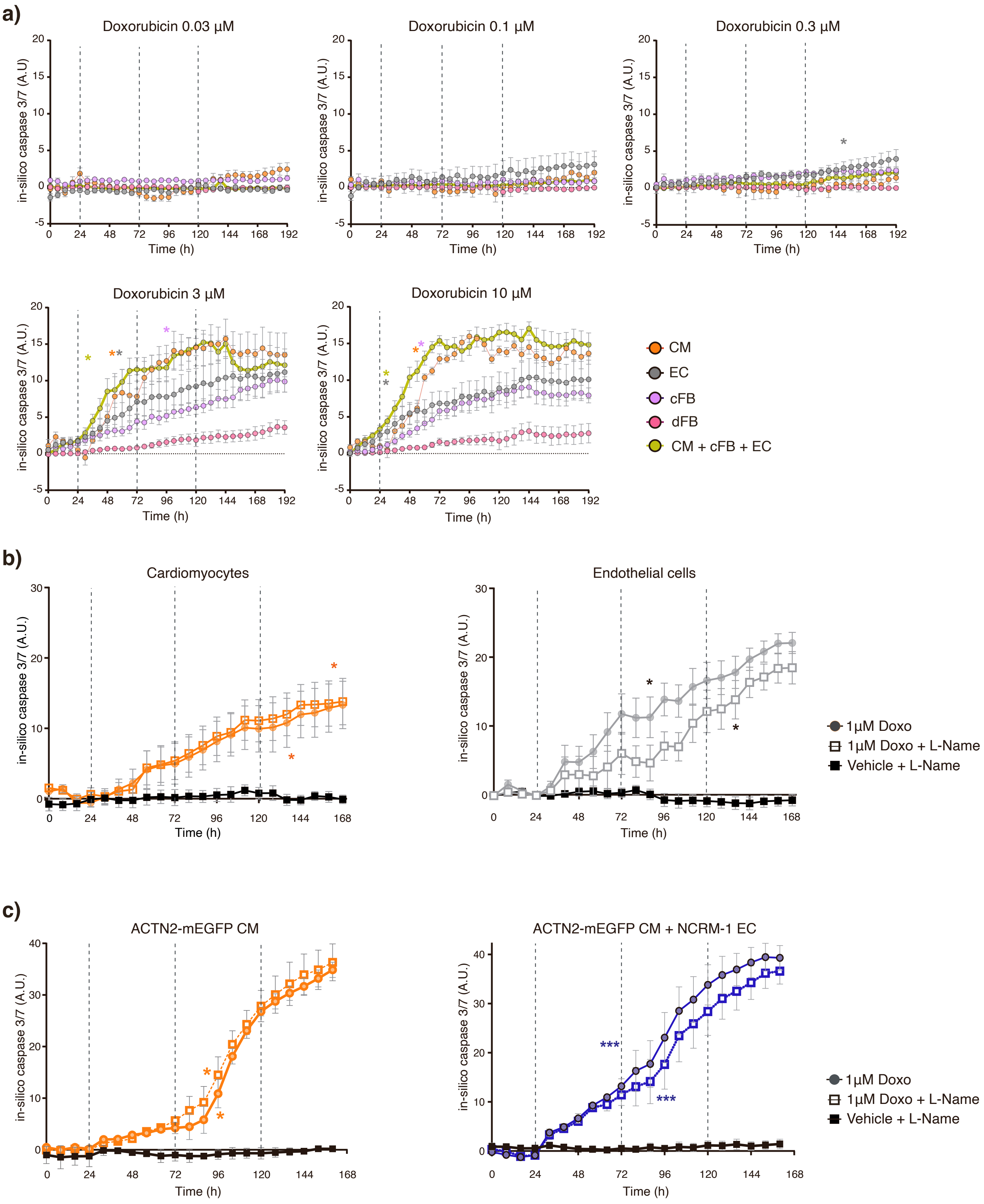

a)
Doxorubicin 0.03 µM
Doxorubicin 0.1 µM
Doxorubicin 0.3 µM
Doxorubicin 3 µM
Doxorubicin 10 µM
in-silico caspase 3/7 (A.U.)
Time (h)
CM
EC
cFB
dFB
CM + cFB + EC
b)
Cardiomyocytes
Endothelial cells
in-silico caspase 3/7 (A.U.)
Time (h)
1µM Doxo
1µM Doxo + L-Name
Vehicle + L-Name
c)
ACTN2-mEGFP CM
ACTN2-mEGFP CM + NCRM-1 EC
in-silico caspase 3/7 (A.U.)
Time (h)
1µM Doxo
1µM Doxo + L-Name
Vehicle + L-Name

Figure S5. *In silico* quantification of caspase 3/7 activity in isogenic hiPSC-derived cells treated with Doxo and L-Name, related to Figures 4 and 5.

a) *In silico* quantification of caspase 3/7 activity in isogenic monocultures and in multi-cell type culture condition (hiPSC-CMs, -ECs, and -cFBs) treated with various concentrations of Doxo. All treatments (dotted line) followed the cumulative treatment protocol outlined in Fig. 1, except 10 μM Doxo, which was administered as a single exposure.

b) *In silico* quantification of caspase 3/7 activity in hiPSC-CM monocultures (left) and hiPSC-EC monocultures (right) treated with 1 μM Doxo in the presence or absence of 100 μM L-NAME.

c) *In silico* quantification of caspase 3/7 activity in independent sets of hiPSC-differentiated cells: α-actinin-mEGFP hiPSC-CMs (ACTN2-mGFP) in monoculture (left) or co-cultured with NCRM-1 hiPSC-ECs (right) and treated with 1 μM Doxo in the presence or absence of 100 μM L-NAME.

Caspase 3/7 activity was normalized to the corresponding vehicle control for each culture condition. Color-coded asterisks indicate the first time point at which caspase 3/7 activity was significantly higher than baseline (0 h) for each cell type. Statistical significance was determined using two-way repeated measures ANOVA with Sidak's multiple-comparison test. Data represent mean ± SEM (n = 3 biological replicates, each with 3 technical replicates).

Table S1. Antibody information, related to Methods.

Antibody	Manufacturer	Identifier	Application
Mouse Anti-Type VII collagen	Sigma Aldrich	C6805; RRID: AB_476860	Immunofluorescence
Mouse Anti-Type I Collagen	Sigma Aldrich	MAB3391; RRID: AB_94839	Immunofluorescence
Rabbit Anti-Vimentin	Thermo Fisher Scientific	PA5-27231; RRID: AB_2544707	Immunofluorescence
Donkey anti-Rabbit IgG (H+L) Highly Cross-Adsorbed Secondary Antibody, Alexa Fluor™ Plus 647	Thermo Fisher Scientific	A32795; RRID: AB_2762835	Immunofluorescence
Donkey anti-Mouse IgG (H+L) Highly Cross-Adsorbed Secondary Antibody, Alexa Fluor™ Plus 555	Thermo Fisher Scientific	A32773; RRID: AB_2762848	Immunofluorescence
PE mouse anti-human CD73	BD Pharmingen	561014; RRID: AB_2033967	Flow cytometry
PE-Cy5 mouse anti-human CD90	BD Pharmingen	561972; RRID: AB_10898004	Flow cytometry
FITC anti-human Vimentin REAfinity	Miltenyi Biotec	130-116-663; RRID: AB_2727645	Flow cytometry
VioBlue anti-human Cardiac Troponin I REAfinity	Miltenyi Biotec	130-120-402; RRID: AB_2783891	Flow cytometry
Alexa 488 anti-human CD144 (VE Cadherin)	Thermo Fisher Scientific	53-1449-42; RRID: AB_10753926	Flow cytometry

Table S2. List of compounds tested, related to Methods.

Compound	Manufacturer	Catalog number	Stock concentration (mM)	Final concentration (µM)
Isoprenaline	Sigma	I5627	10	1
Levosimendan	Merck	141505-33-1	100	0.3
Milrirone	Tocris Bioscience	1504	100	100
Bay K 8644	Tocris Bioscience	1544	100	1
Omecamptiv	Sigma	S2623	1	0.3
CDN1163	Tocris Bioscience	1163	100	10
Oubain	Tocris Bioscience	1076	100	3
Doxorubicin	SelleckChem	S1208	100	0.03 - 10
Nifedipine	Tocris Bioscience	1075	100	0.3
Sorafenib	Tocris Bioscience	6814	20	10
Blebbistatin	Sigma	B0560	10	1
Carfilzomib	SelleckChem	S2853	1	3
DMSO	Sigma	D2650	100	3 - 100
L-NAME	Sigma	N5751	10	100

Supplemental Methods

hPSC culture and differentiation

The medical ethics committee (Leiden University Medical Center) approved the use of hPSC lines in this study (P13.080). All cell lines were routinely tested for mycoplasma using the MycoAlert® Mycoplasma Detecton Kit (Lonza, #LT07-318), and the identity of the cell lines confirmed either by STR analysis (Idexx BioAnalytics) or by visualization of the genetically tagged fluorescent reporters.

The hPSC lines were differentiated into hPSC-CMs using a small molecule protocol [S1]. Briefly, the hPSCs were seeded on Matrigel (Corning)-coated 12 well plates at either 7.5 x 10^4 (HES-3 MESP1^{mcherry}-NKX2.5^{eGFP}), 1.5 x 10^5 (LUMC0020iCTRL-06) or 2.5 x 10^5 (alpha-actinin-2^{mEGFP}) cells/well 24 h (day -1) prior to starting the differentiation. On differentiation day 0, the medium was replaced with mBEL supplemented with 5 µM CHIR99021 (Axon Medchem), followed by mBEL supplemented with 5 µM XAV939 (Tocris Bioscience) and 0.25 µM IWP-L6 (Axon Medchem) on day 2. On day 4, medium was changed to mBEL supplemented with insulin-transferrin-selenium-ethanolamine (ITS-X; Thermo Fisher Scientific). The medium was then replaced every 2-3 days with CM Specification Medium (mBEL minus essential lipids) until the cells were harvested for cryopreservation (differentiation day 14 unless otherwise indicated).

hiPSC-EC differentiation was performed as previously described [S1, S2], with minor modifications. The hiPSCs (LUMC0020iCTRL-06 or NCRM-1) were seeded on Matrigel-coated wells 1 day prior to starting the differentiation. Mesoderm formation was induced in mBEL supplemented with 5 µM CHIR 99021 or B(P)EL medium containing 8 µM CHIR 99021. On differentiation day 3, the medium was replaced with vascular specification medium (either mBEL + ITX-S containing 50 ng/ml VEGF (R&D Systems), or B(P)EL + 50 ng/ml VEGF + 10 µM SB431542 (Tocris Bioscience)), and subsequently refreshed every 2-3 days. The hiPSC-ECs were isolated using either EasyStep™ CD34 Human Cord Blood Isolation Kit II (STEMCELL Technologies) or CD31-Dynabeads™ (Thermo Fisher Scientific) and expanded as previously described for cryopreservation [S1, S2].

The hiPSCs (LUMC0020iCTRL-06) were differentiated into hiPSC-cFB via epicardial progenitor cells [S1], with the modification that the hiPSCs were seeded at 2.0 x 10^4 cells/cm^2 and 5 µM CHIR99021 was used for mesoderm induction (differentiation day 0 to 2). On day 2, the medium was replaced with mBEL supplemented with 5 µM XAV, 1 µM Retinoic Acid (Sigma Aldrich) and 30 ng/ml BMP4 (R&D Systems). From day 4, BMP4 was removed from the culture medium. On differentiation day 9, the epicardial cells were seeded on fibronectin (5 µg/ml bovine plasma; Sigma Aldrich)-coated wells in mBEL supplemented with 10 µM SB431552. When confluent, the epicardial cells were either cryopreserved or further differentiated to cFB by seeding on vitronectin-coated plates and culturing for 6 days in mBEL supplemented with 10 ng/ml FGF2 (R&D Systems). The cFBs were subsequently expanded in Fibroblast Growth Medium 3 (PromoCell), with medium refreshment every 2-3 days until harvested for passaging or cryopreservation.

The hiPSC-dermal fibroblast (hiPSC-dFB) differentiation was essentially as previously described [S3]. Briefly, hiPSCs (LUMC0020iCTRL-06) were aggregated (3.5 x 10^4 cells/well in 100 mL TeSR-E8 + 10 mM Y27632 (Stemgent)) in U-bottom low-attachment 96-well plates (Thermo Fisher Scientific) by centrifugation at 110*g* for 6 min. After 24 h (differentiation day 0), aggregates were collected, washed 3 times with E6 medium (Thermo Fisher Scientific) and transferred to a new U-bottom low-attachment 96-well plate in 100 µL dFB-differentiation medium (E6 medium containing 10 µg/mL TGFb2 (R&D Systems), 0.1 mM L-ascorbic acid (Merck), 1x ITS-A supplement (Thermo Fisher Scientific) and 100 µg/mL Normocin (Invivogen)). After 24 h (differentiation day 1), an additional 50 µL dFB-differentiation medium added to each well. On differentiation day 3, 15-20 aggregates were transferred to each well of a 6-well plate coated with 0.1% gelatin (Sigma Aldrich) and containing DMEM/F12 medium supplemented with 1x GlutaMAX, 20% Fetal Bovine Serum (all Thermo Fisher Scientific) and 0.1 mM L-ascorbic acid. On day 13, cells were harvested using 1x TrypLE Select enzyme (Thermo Fisher Scientific) and replated (dilution 1:3) in dFB-maintenance medium (DMEM/F12 medium + 1x GlutaMAX, 10% Fetal Bovine Serum, 1x non-essential amino acids, 50 U/mL Penicillin, 50 mg/mL Streptomycin, 0.09 mM 2-mercaptoethanol (all Thermo Fisher Scientific), 0.1 mM L-ascorbic acid). The cells were replated at a 1:5 ratio and maintained in dFB-maintenance medium with medium changed every 2 days until wells were confluent for passaging or cryopreservation. Cells were passaged a maximum of 5 times.

All experiments were performed with cryopreserved, differentiated hPSCs. Briefly, thawed hPSC-CMs were cultured on Matrigel-coated wells in mBEL CM Maintenance Medium [S1], with RevitaCell™ Supplement (1:200 dilution) added for the first 24 h to improve recovery. Thawed hiPSC-ECs were cultured on fibronectin-coated wells in mBEL vascular specification medium, while thawed hiPSC-cFB and hiPSC-dFB were cultured in FGM3 or dFB-maintenance medium, respectively, on uncoated wells. The purity of the cryopreserved cell types was evaluated based on criteria previously

described [S1, S3]. Only hPSC-CM batches with >85% cTnT$^+$ cells, hiPSC-cEC batches with >85% CD144 (VE-cadherin)$^+$ cells, hiPSC-cFB batches with >85% Vimentin$^+$ cells, and hPSC-dFB batches with >90% CD73$^+$ and CD90 (Thy-1)$^+$ cells, as determined by flow cytometry, were used.

Between 5-7 days after thawing all cells were harvested using 1x TrypLE Select enzyme, except for the hPSC-CMs which were harvested using 5x TrypLE Select solution. The cells were seeded in 96-well plates. For monocultures, either 6 x 10^4 hPSC-CMs, 5 x 10^4 hiPSC-ECs, 1 x 10^4 hiPSC-cFBs or hiPSC-dFBs were seeded in each well, so that cell confluencies were similar at the start of the experiment. For multi-cell type cultures, a total of 6 x 10^4 cells plated per well. For cultures containing 3 cell types, the cell composition was 70% hPSC-CMs, 15% hiPSC-ECs, and 15% hiPSC-cFBs or -dFBs, with the cells cultured in mBEL CM Maintenance Medium + 50 ng/ml VEGF and 5 ng/ml FGF2, or a 1:1 mix of mBEL CM Maintenance Medium + 50 ng/ml VEGF and dFB Maintenance Medium, respectively. Cultures containing 2 cell types consisted of 85% hPSC-CMs with either 15% hiPSC-ECs in mBEL CM Maintenance Medium + 50 ng/ml VEGF, or 15% hiPSC-cFBs in mBEL CM Maintenance Medium + 5 ng/ml FGF2.

Flow Cytometry

Single cell suspensions were prepared by washing the cells once with FACS buffer (1× PBS, 0.5% BSA, 2 mM EDTA) and subsequently filtering the suspension. When necessary, cells were fixed and permeabilized using the Fix & Perm Cell Permeabilization Kit (Invitrogen) following the manufacturer's instructions. Cells were incubated with the antibodies listed in **Table S1**. Measurements were acquired using a MACSQuant VYB (Miltenyi Biotec) or LSR II (BD Biosciences) flow cytometer, and data analyzed using FlowJo software (FlowJo, LLC).

Immunofluorescence

Cells were fixed in 1% paraformaldehyde in PBS for 30 min at room temperature (RT). Permeabilization and blocking were performed for 1 h at RT in PBS containing 0.1% Triton X-100 and 4% normal swine serum (Jackson ImmunoResearch, #014-000-121). Cells were incubated with the primary and the appropriate fluorophore-conjugated secondary antibodies (**Table S1**) diluted in PBS for 1 h at RT. Washes were performed with PBS + 0.05% Tween. Nuclei were stained with DAPI (Fisher Scientific, #D3571) and images acquired using a SP5 confocal microscope (Leica).

Cumulative Doxo treatment

For the cumulative treatment protocol, Doxo was diluted to the required concentration in the culture medium appropriate for each cell type. The culture medium on the cells was replaced with 100 µL Doxo-containing medium for 4 h at 37°C, 5% CO_2. Post incubation, the medium was removed and replaced with the standard culture medium. This cycle was repeated 3 times at 48 h intervals for all concentrations of Doxo, except 10 µM which was only incubated with the cells once. DMSO vehicle control concentration (0.1% v/v) matched that of 3 µM Doxo, which was the highest concentration used in the cumulative treatment protocol. Additionally for comparing toxicity, hiPSC-dFBs were treated with 3 µM carfilzomib following an identical protocol and timing.

For NOS-inhibition experiments, L-NAME (100 µM; Sigma #N5751) was included in the treatment medium during each 4 h exposure to Doxo or DMSO vehicle and re-added at every medium change, so that it remained present throughout the entire cumulative treatment period.

Live cell imaging and fluorescence analysis

To detect apoptosis, cells were labelled with either the red or green Incucyte Caspase 3/7 dyes for apoptosis (Sartorius) according to manufacturer's instructions. Briefly, the cells were treated with the Caspase 3/7 reagent at final concentrations of 2.5 µM (red) or 5 µM (green) diluted in the appropriate cell culture medium (100 µL per well), 1 h before Doxo treatment. Initial baseline fluorescence images were obtained prior to drug administration. After drug exposure, the medium was replaced with fresh culture medium containing the Caspase 3/7 dye. This process was repeated at 48 h intervals. Fluorescence imaging using either the red (500 ms acquisition) or green (300 ms acquisition) channels, along with phase contrast imaging, was conducted at regular 3 h intervals.

To analyze phase confluence, eGFP (NKX2.5) expression and caspase 3/7 signal, the Incuycte's integrated software (Incucyte® 2022B Rev2) was used, employing either classic or AI-enhanced confluency modes for phase contrast, and Surface Fit or Top-Hat for fluorescence segmentation depending on the fluorescent channel. Measurements were

normalized to the initial time point (t0) or to phase contrast area for comparative analysis. No image or well was excluded from analysis.

Generation of *in silico* models

Deep neural network (DNN) models were generated to identify hPSC-CMs within a triple culture system and to detect caspase 3/7 activity from phase contrast images. For cardiomyocyte identification, phase contrast images acquired using the Incucyte® system were paired with corresponding fluorescence images depicting NKX2.5 expression, which served as ground truth. Fluorescence images were preprocessed prior to use in model training.

Image segmentation was performed using a U-Net–based architecture [S4] incorporating skip connections to preserve spatial information. The cardiomyocyte model was trained on a dataset comprising 2,500 paired phase contrast and fluorescence images collected over nine consecutive days.

A second DNN model was trained to detect caspase 3/7 activity using a similar workflow. Both models were trained using SmoothL1Loss over 15 epochs with a batch size of 4. The NKX2.5 model utilized a learning rate of 0.00001, while the Caspase model employed 0.0001. The performance of each model was evaluated by comparing the accuracy of the predicted labelling to actual labelled images (NKX2.5: 7200 analyzed images; Caspase: 8537 analyzed images) not used in the training of the model.

in silico image analysis and data processing

Archives of the phase contrast images, which included imaging data collected throughout the experiment, along with detailed plate map of conditions and preliminary analyses conducted using the Incucyte® system's integrated software (basic well confluency), were exported from the Incucyte®. No image or well was excluded from the analysis.

This data was subsequently analyzed with the caspase 3/7 and NKX2.5 DNN tools (Sartorius) using Python programming language and the Pytorch framework for deep learning. *In silico* caspase 3/7 output data was further processed in R (version 4.3.2). For each time point and cell type, DMSO vehicle control values were averaged across 3 technical replicate wells and subtracted from individual caspase 3/7 measurements. The resulting values were normalized to the initial time point (0 h).

The *in silico* NKX2.5$^+$ values were normalized by dividing each time point by the corresponding initial value (0 h). Normalization and correction procedures were performed separately for each biological replicate.

Supplemental References

[S1] Campostrini, G., Meraviglia, V., Giacomelli, E., van Helden, R.W.J., Yiangou, L., Davis, R.P., Bellin, M., Orlova, V.V., and Mummery, C.L. (2021). Generation, functional analysis and applications of isogenic three-dimensional self-aggregating cardiac microtissues from human pluripotent stem cells. Nat. Protoc. *16*, 2213-2256. 10.1038/s41596-021-00497-2.

[S2] Orlova, V.V., van den Hil, F.E., Petrus-Reurer, S., Drabsch, Y., Ten Dijke, P., and Mummery, C.L. (2014). Generation, expansion and functional analysis of endothelial cells and pericytes derived from human pluripotent stem cells. Nat Protoc *9*, 1514-1531. 10.1038/nprot.2014.102.

[S3] Itoh, M., Umegaki-Arao, N., Guo, Z., Liu, L., Higgins, C.A., and Christiano, A.M. (2013). Generation of 3D skin equivalents fully reconstituted from human induced pluripotent stem cells (iPSCs). PLoS One *8*, e77673. 10.1371/journal.pone.0077673.

[S4] Falk, T., Mai, D., Bensch, R., Çiçek, Ö., Abdulkadir, A., Marrakchi, Y., Böhm, A., Deubner, J., Jäckel, Z., and Seiwald, K. (2019). U-Net: deep learning for cell counting, detection, and morphometry. Nat. Methods *16*, 67-70. 10.1038/s41592-018-0261-2.
